# Advances in Pretreatment of Straw Biomass for Sugar Production

**DOI:** 10.3389/fchem.2021.696030

**Published:** 2021-06-07

**Authors:** Jinyu Tan, Yan Li, Xiang Tan, Hongguo Wu, Hu Li, Song Yang

**Affiliations:** ^1^ State Key Laboratory Breeding Base of Green Pesticide and Agricultural Bioengineering, Key Laboratory of Green Pesticide and Agricultural Bioengineering, Ministry of Education, State Local Joint Engineering Laboratory for Comprehensive Utilization of Biomass, Center for R&D of Fine Chemicals, Guizhou University, Guiyang, China; ^2^ Institute of Crops Germplasm Resources, Guizhou Academy of Agricultural Sciences, Guiyang, China

**Keywords:** straw biomass, pretreatment, fermentation, saccharification, enzymatic hydrolysis

## Abstract

Straw biomass is an inexpensive, sustainable, and abundant renewable feedstock for the production of valuable chemicals and biofuels, which can surmount the main drawbacks such as greenhouse gas emission and environmental pollution, aroused from the consumption of fossil fuels. It is rich in organic content but is not sufficient for extensive applications because of its natural recalcitrance. Therefore, suitable pretreatment is a prerequisite for the efficient production of fermentable sugars by enzymatic hydrolysis. Here, we provide an overview of various pretreatment methods to effectively separate the major components such as hemicellulose, cellulose, and lignin and enhance the accessibility and susceptibility of every single component. This review outlines the diverse approaches (e.g., chemical, physical, biological, and combined treatments) for the excellent conversion of straw biomass to fermentable sugars, summarizes the benefits and drawbacks of each pretreatment method, and proposes some investigation prospects for the future pretreatments.

## Introduction

Lignocellulosic biomass is usually composed of agriculture residues (rice straw, corn straw, wheat straw, risk husk, sugarcane bagasse, cotton straw, and other plant residues), forest residues (wood), industrial residues (pulp and paper processing waste), and energy crops (switchgrass) (Akhtar et al., 2016; Chen et al., 2017; Kumar and Sharma, 2017; Hassan et al., 2018). Generally, straw biomass is one of the agriculture residues, which is abundant, inexpensive, clean, safe, renewable, and sustainable, and can alleviate the contradiction in applications between energy and food, which serves as the best selection to replace conventional fossil energy resources (Steinbach et al., 2017). Most of the straw biomass could be transformed into numerous forms of high-value chemicals, which can reduce the environmental issues, and facilitate the sustainable development of economics and society (Kim et al., 2012). Lignocellulosic biomass is principally composed of cellulose, hemicellulose, and lignin, in which fermentable sugars are achieved by hydrolysis of sugar components (Tian et al., 2018). However, numerous hurdles are associated with efficient application due to the complex compositions that are strongly connected in diverse straw biomass.

**FIGURE 1:**
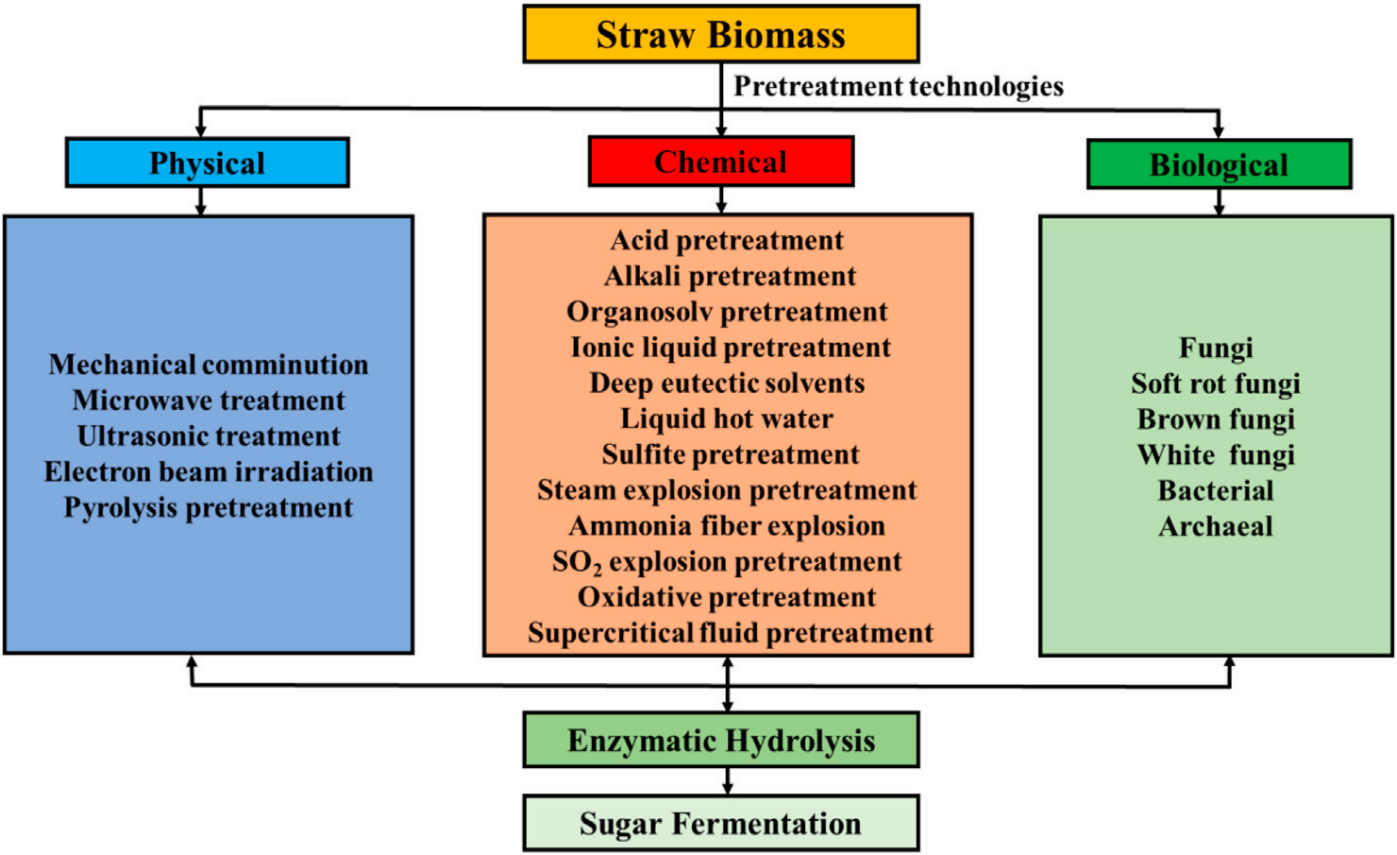
Summary of different methods for pretreatment of lignocellulosic biomass.

Plenty of straws such as corn stover/cob (1,661 million tons), wheat straw (529 million tons), and rice straw (975 million tons) are produced every year in the world (Hilares et al., 2017). In China, 1 billion tons of straw biomass are obtained each year (Zhong et al., 2011). The production of straw has increased at a rate of 1.4% annually (Zeng et al., 2007). Approximately 81.48% of crop straw could be used in China (Qiong et al., 2019). Only elevated 20 times of the environmental efficiency of existing agricultural production technologies such as the utilization of energy, space, and raw materials can probably realize sustainable development in 2040 (Singh et al., 2016). However, the majority of straw biomass is directly burned, unused, and discarded, leading to resource waste, environmental pollution, and ecosystem problems (Kim and Dale, 2004; Li et al., 2018; Yu et al., 2019). Hence, it is crucial and urgent to design appropriate pretreatment methods, which can effectively increase the utilization of feedstock and decrease its cost to obtain energy and environmental benefits (Kapoor et al., 2017). Diverse treatment methods are summarized in Figure 1.

Current research on treatment technologies primarily concentrates on identification, estimation, development, and demonstration for subsequent enzymatic digestion that needs less conversion time and low enzyme dosage. Suitable pretreatment approaches should be focused on the highest fermentable sugars with the lowest inhibitors and elevate the efficiency of the overall process, which involves pretreatment, enzymatic digestion, and fermentation. Remarkably, every step reveals its hurdles that increase costs of the total treatment process. Therefore, each step is very crucial to obtain ideal results (Bhaskar et al., 2016).

Plenty of criteria are needed for the choice of an appropriate pretreatment technique: 1) effectively disrupting the complexly interlinked fraction components, 2) enhancing cellulose accessibility and lignin removal, 3) preserving hemicellulose fraction as much as possible, 4) decreasing the solubility of lignin and increasing the recovered purity of lignin, 5) reducing the loss of cellulose and improving enzymatic digestion efficiency, 6) minimizing the side products, 7) reducing the energy consumption, and 8) producing green, safe, and sustainable target products. The objective of this review is to introduce and evaluate different methods developed for the pretreatment of straw biomass to produce fermentable sugars.

### Composition of Straw Biomass

**FIGURE 2:**
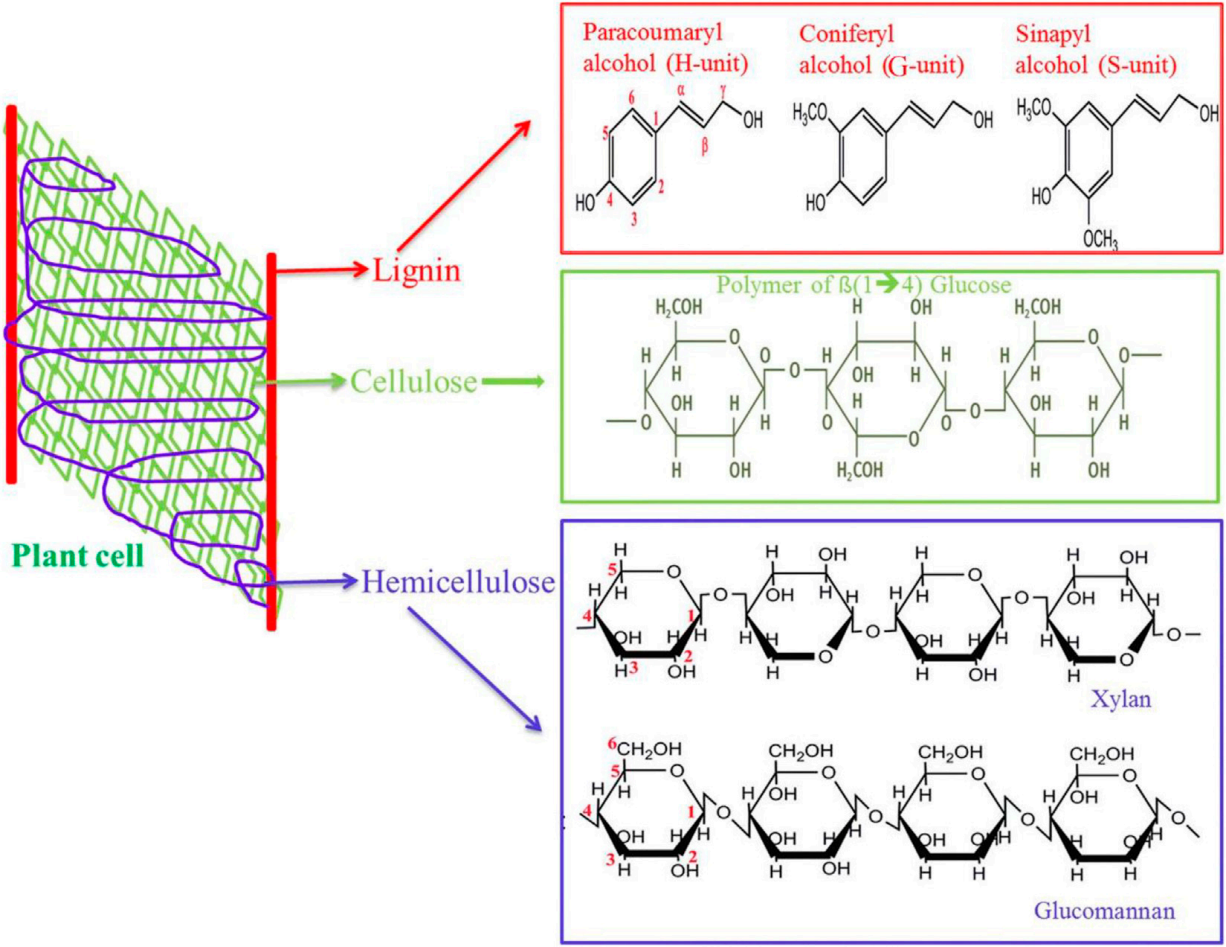
Structural arrangement of straw biomass. Reproduced with permission from Machineni et al. (2019).

Straw biomass is mainly composed of cellulose (40–50%), hemicellulose (25–30%), and lignin (15–20%), which cross each other in space and build a complex network. Physical protection formed via lignin and hemicellulose around cellulose leads to cellulose that is difficult to be hydrolyzed (Figure 2) (Tan et al., 2020). In addition, straw biomass contains a small amount of pectin, fat, nitrogen compounds, inorganic ingredients, and other extracts (Chen et al., 2017).

Cellulose, a major part of the cell wall of plants, is deemed the most enriching natural compound in the world. It is a linear chair polymer and composed of β-1,4-polyacetal of cellobiose (4-O-β-D-glucopyranosyl-D-glucose). Crystalline cellulose is difficult to dissolve into water and a universal organic solvent due to its high degree of crystallinity and polymerization (Karimi et al., 2019; Meneses et al., 2020). Hemicellulose is a stereo-irregular polysaccharide and the second largest polymeric carbohydrate, which consists of different polymeric carbohydrates that have low polymerization and no crystalline regions. Therefore, hemicellulose is easily transformed into monosaccharides such as xylan, xyloglucan, arabinogalactan, galactoglucomannan, and glucomannan. Lignin is the third largest inexhaustible natural polyphenolic compound after cellulose and represents the major natural aromatic resource (Holmgren et al., 2006; Zakzeski et al., 2010; Ponnusamy et al., 2019). It is composed of phenyl propane including three unit compounds: sinapyl alcohol, coniferyl alcohol, and *p*-coumaryl alcohol, which connects with ester bonds and carbon–carbon bonds forming a complex network to prevent the enzymatic hydrolysis of cellulose (Akhtar et al., 2016). In addition, pectin is located in the cell wall and middle lamella of plants, which acts as a main plant load–bearing component and plays a “glue” role in holding cell-wall components together (Chen et al., 2017; Satari et al., 2019). Furthermore, lignin has strong hydrophobicity. Due to the different structures of hemicellulose, cellulose, and lignin, the transformation of any components could affect the other ingredients’ degradation. Hence, to elevate cellulose accessibility and enzymatic hydrolysis efficiency, some suitable treatment approaches should be utilized to remove or dissolve hemicellulose and lignin (Tan et al., 2020). Different straw materials exhibit various characteristics such as complexity and heterogeneity; therefore, diverse lignocellulosic feedstocks have different composition concentrations (Table 1) (Alinia et al., 2010; Castro et al., 2011; López-Linares et al., 2013; Zhang et al., 2013; Singh and Dhaka, 2015; Bhaskar et al., 2016; Hilares et al., 2017; Kumar and Sharma, 2017; Xu et al., 2017; Wang et al., 2018).

**TABLE 1:** Chemical components of diverse straw biomass (% dry basis).

Source	Cellulose (%)	Hemicellulose (%)	Lignin (%)	References
Mustard straw	32.7–48.3	14.7–29.6	17.7–24.6	Singh et al. (2021)
Corn stover	30–38	26–26.1	11–19	Zhao et al. (2017)
Corn stalk	29.08–35.3	24.1–25.99	13.6–15.04	Wang et al. (2018)
Rice straw	32–47	19–27	5–24	Bhaskar et al. (2016)
Cotton straw	38.7	23.5	23.5	Yildirim et al. (2021)
Wheat straw	35–45	20–30	8–15	Alinia et al. (2010)
*Miscanthus*	40–60	20–40	10–30	Zhang et al. (2021)
Sugarcane peel	41.11	26.4	24.31	Kumar and Sharma (2017)
Sweet sorghum	45	27	21	Kumar and Sharma (2017)
Rapeseed straw	35.5–36.6	22.9–24.1	15.6–16.8	Castro et al. (2011)
Barley straw	35.4	28.7	13.1	Kumar and Sharma (2017)
Rye	42.38	27.86	6.51	Kumar and Sharma (2017)
Sunflower	34.06	5.18	7.72	Kumar and Sharma (2017)

**FIGURE 3:**
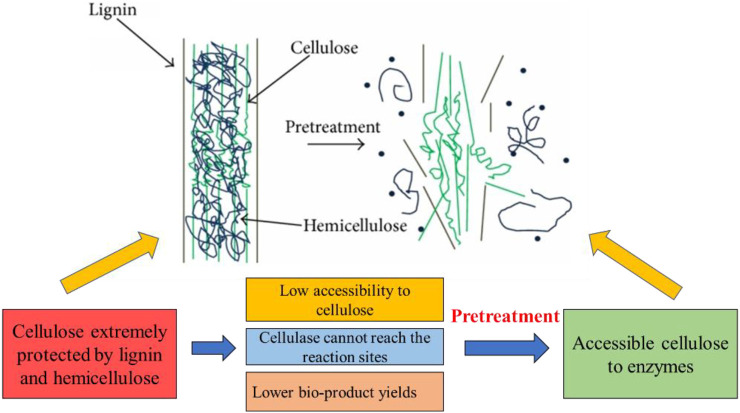
Schematic illustration of biomass pretreatment. Reproduced with permission from Lee et al. (2014).

Before pretreatment of biomass, cellulose was extremely protected by hemicellulose and lignin and resulted in low accessibility of cellulase, which cannot reach the reaction active sites and produce lower object products. In sharp contrast, after pretreatment of biomass, the physical barrier is broken and the hydrogen bonds cracked between hemicellulose and lignin dramatically improved cellulose accessibility and enzymatic hydrolysis (Figure 3) (Morais et al., 2015; Zhao et al., 2018). Hence, only using the suitable pretreatment technologies of different lignocellulosic biomass could effectively enhance the process of saccharification and fermentation.

## Utilization of Straw Biomass

Biomass is carbon-neutral, which is a renewable and sustainable organic carbon source with zero carbon emissions (Weerasai et al., 2014; Ullah et al., 2015). The fermentable sugars could be obtained from cellulose and hemicellulose via many suitable pretreatment technologies (Nguyen et al., 2010; Ullah et al., 2015). Therefore, the process of conversion of lignocelluloses to fermentable sugars is the crucial step to convert straw biomass into other valuable chemicals and biofuels (Wang et al., 2018; Meneses et al., 2020).

**FIGURE 4:**
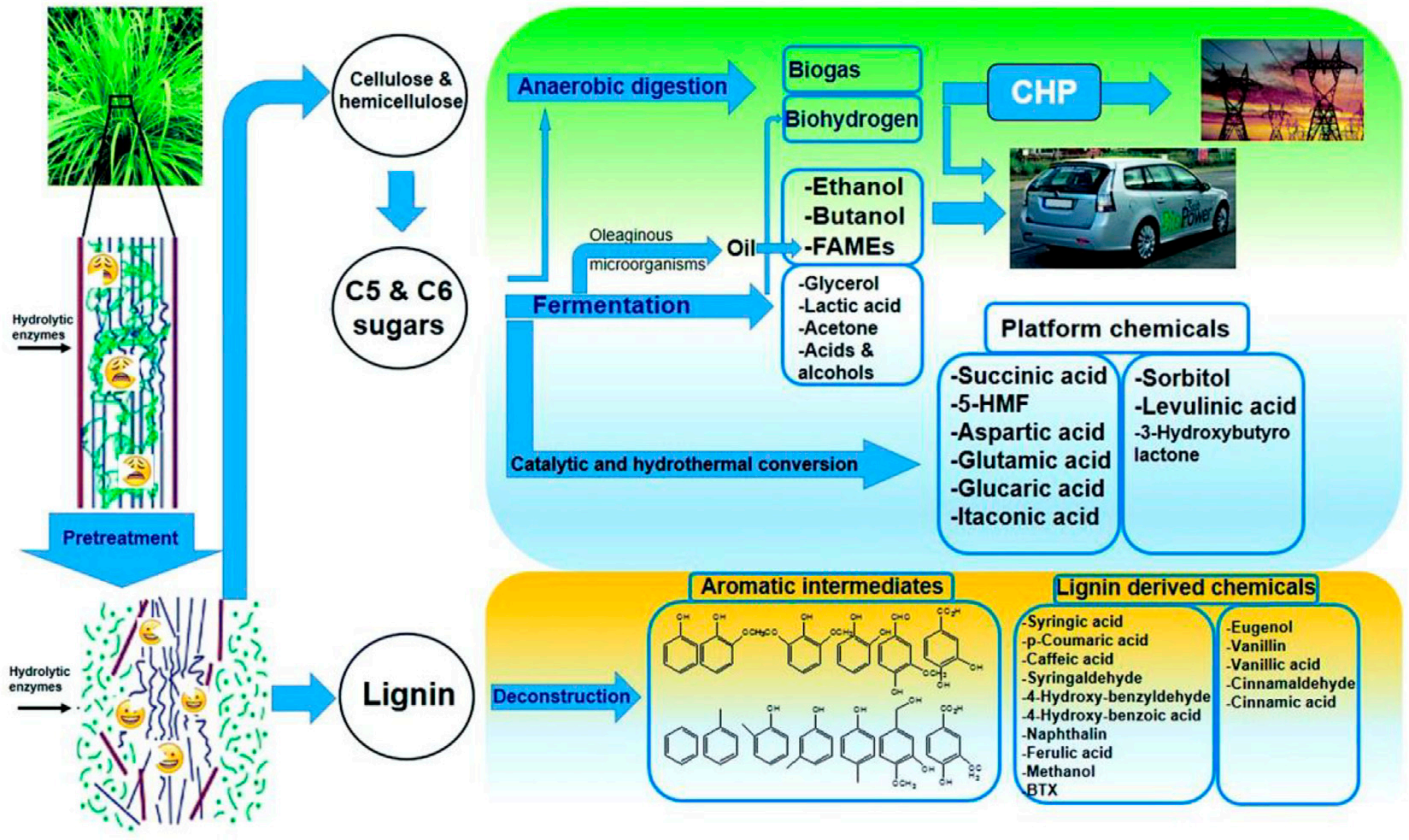
Generation of diverse valuable chemicals and biofuels from lignocellulosic biomass. Reproduced with permission from Satari et al. (2019).

At present, the range of applications becomes wider, such as direct combustion, anaerobic digestion, straw gasification, straw briquette, and others. Furthermore, sugar or ethanol could be obtained from straw biomass through pretreatment, fermentation, hydrolysis, and syngas that could be produced from the gasification of residue, which could be further transformed into liquid biofuels under the action of the catalyst (Ullah et al., 2015). In addition, anaerobic fermentation would dramatically facilitate the degradation of lignocellulosic materials because the relationship between lignin and polysaccharides was broken down and cellulose and hemicellulose were easier to be digested by bacteria (Song et al., 2014). Nevertheless, fermentative hydrogen production from straw is highly meaningful because it can produce clean energy (H_2_) and also reduce pollution caused by traditional burning (Yuan et al., 2020). In conclusion, straw biomass can be converted into various valuable chemicals and biofuels via diverse pretreatment techniques (Figure 4).

## Chemical Pretreatment

### Liquid Hot Water

Liquid hot water (LHW) treatment is an efficient and environmentally friendly technology (Min et al., 2015). LHW treatment is also called solvolysis, hydrothermolysis, aqueous fractionation, and aquasolv, which employs water to treat biomass at high pressure (up to 5 MPa) and temperature (200 ± 20°C) (Kumar and Sharma, 2017; Wang et al., 2018) so that water maintains its liquid state and acts as an acid. LHW treatment attracts increasing attention because it does not need a supplement of chemicals or catalysts and quick-release pressure or expansion. Moreover, it generates only less inhibitors as compared to steam explosion (SE), with the lower cost of treatment reactors, retaining the high production of sugars, almost neutral pH value, and lower corrosion. In addition, based on the hydrolysis rates of hemicellulose and cellulose being distinguished, a two-stage LHW treatment could be designed. However, the major shortcomings of the LHW process require a rigorous reactor configuration (Oliveira et al., 2014; Capolupo and Faraco, 2016; Wang et al., 2018). Besides, it would consume a large amount of energy in downstream processing because plenty of water is included (Rajput and Zeshan Visvanathan, 2018; Shahabazuddin et al., 2018; Yang et al., 2018).

LHW treatment can degrade 80% hemicellulose of diverse feedstocks such as wheat straw and corn stover (Capolupo and Faraco, 2016). Wheat straw was pretreated by two-stage hydrothermal processes and subsequent enzymatic hydrolysis to achieve 66% total recovery of sugars and the lower content of byproducts (Min et al., 2015). Furthermore, the two-stage LHW treatment of corn stover achieved 89.55% recovery of glucose after 72 h enzymatic hydrolysis. In addition, one-stage LHW pretreatment of acetic acid–rich spent liquor has been studied as compared to two-stage LHW treatment. As a result, 89.55% of glucose was obtained by utilizing acetic acid–rich spent liquor treatment, while 80.58% of glucose was obtained by applying one-stage LHW treatment (Lü et al., 2017). Therefore, to gain maximum fermentable sugars, a two-stage treatment was the most suitable technology (Pérez et al., 2008). Importantly, LHW and alkaline soaking were applied for the treatment of soybean straw at ambient temperature, respectively, both furnishing almost 100% cellulose under the treatment approaches. The yield of glucose was obtained up to 64.55% and xylan was removed up to 46.37% with NaOH soaking at ambient conditions, while 70.76% glucose was achieved and xylan was removed up to 80% at 210°C for 10 min by LHW (Wan et al., 2011).

Although LHW treatment is a promising approach for converting straw biomass to fermentable sugars, the optimum treatment conditions are difficult to design for various lignocellulosic biomass. Furthermore, investigations have revealed various efficiency of LHW pretreatment conditions from different lignocellulosic biomass to fermentable sugars (Rezania et al., 2020). To predict the optimum reaction condition that could obtain the highest sugar yield from different biomass, the general additive models (GAMs) were applied to visualize LHW pretreatment on Napier grass and energycane and achieved the highest glucose yield (Wells et al., 2020). Therefore, it is a promising method that applies certain models (GAMs) to improve the LHW pretreatment efficiency.

## Alkali Pretreatment

Alkali pretreatment is an efficient and cost-effective approach to generate fermentable sugars, which is to mainly swell the raw materials through degrading the ester bonds and glycosidic bonds in the cell wall of lignocellulose (Das et al., 2021). It can remove lignin and hemicellulose and increase the surface area and porosity of pretreated straw biomass. Besides, it can effectively disrupt the structure of lignin, elevate the accessibility of cellulose, decrease the crystallinity and polymerization of cellulose, and improve the polysaccharides’ activity (Nargotra et al., 2018). This pretreatment only requires low temperature (<100°C) and low alkali concentration (<2%) depending on various lignocellulosic feedstocks (Yu et al., 2010).

The alkaline loading, reaction time, and temperature are the main effective factors of lignin removal and fermentable sugar production. Among them, the factors like high alkaline concentration, long reaction time, and high temperature can enhance the efficiency of saccharification and fermentation (Cheng et al., 2010). Utilizing rice straw researched the influence of the sodium hydroxide concentration, temperature, and duration of alkali pretreatment with response surface methodology, and the highest yield of glucose (254.5 ± 1.2 g kg^−1^) could be obtained from enzymatic digestion at the optimal conditions (2.96% sodium hydroxide, 81.79°C, 56.66 min) (Kim and Han, 2012). In addition, with the conjunction of ozone and alkaline treatment of corn straw, the ratio of cellulose hydrolysis reached 91.73% (Travaini et al., 2013). Then, grinding coupled with NaOH pretreatment of wheat straw remarkably altered the surface structure; the content of hemicellulose and lignin was decreased by 44.15 and 42.52%, respectively, and the cellulose content was enhanced by 44.52% under the optimal pretreatment conditions of 120 mashes’ feedstock size, 1.0% of sodium hydroxide concentration, 1.5 h reaction time, 121°C temperature, and 0.1 MPa pressure (Tang et al., 2017). In addition, rice straw was pretreated with 1% NaOH, achieving 61.9% cellulose content and 37.51% lignin content, in comparison with the untreated one only obtaining 52.75% cellulose content and 9.93% lignin content, respectively (Samar et al., 2020).

As an inexpensive treatment approach, it has many advantages such as the lower cost of operation, an inferior yield of sugar degradation, lower energy consumption, lesser corrosion compared to acid treatment, lower content of lignin, and lesser inhibitors (Kim et al., 2014; Cai et al., 2016). However, there are some shortcomings of alkali treatment. For instance, compared to acid treatment, the unrecovered salts were formed or the salts were mixed with the biomass. Therefore, it is difficult to recover the salts formed in the process of pretreatment (Mosier et al., 2005). Alkali pretreatment can not only significantly remove lignin from straw biomass but also degrade some parts of hemicellulose and cellulose. An appropriate and desirable approach could remove lignin as much as possible while preserving the fermentable sugars. Consequently, reaction conditions for alkaline treatment would be designed depending on the lignin removal rate and fermentable sugar yields on enzyme digestion. In general, sulfite, lime, ammonium hydroxide, and sodium hydroxide are usually applied to the pretreatment of straw biomass. Among these chemicals, NaOH is the most popular alkaline because of its excellent delignification efficiency (Rezania et al., 2020).

## Acid Pretreatment

Acid treatment of straw biomass is a common method, mainly through disrupting the linkage between hemicellulose, lignin, and cellulose, improving the hemicellulose hydrolysis efficiency and lignin removal, and further accelerating the saccharification and fermentation processes (Mosier et al., 2005). In the process of pretreatment, inorganic and organic acids are used usually (Chen et al., 2017). Inorganic acid pretreatment involves acid concentration and dilutes acid pretreatment. Dilute acid hydrolysis is required after concentrated acid treatment (Kapoor et al., 2017). Acid treatment can efficiently convert straw biomass into fermentable sugars. Treatment with various acids has revealed discrepant activity in the production of fermentable sugars in diverse materials (Mosier et al., 2005). In the pretreatment of inorganic acids, the utilization of phosphoric acid has many unique characteristics including a lower environmental impact, a nutrient for the fermenting microorganisms, and the best yield parameters. Phosphoric acid with different concentrations for the treatment of various feedstocks exhibited a diverse influence on saccharification (Table 2). Furthermore, concentrated sulfuric and nitric acid pretreatments of lignocellulosic biomass are the most important approaches for commercial utilization (Zhao et al., 2021). However, the strong acid treatment not only requires high-energy input, rigorous equipment, and hazardous chemicals but also generates inhibitors and causes environmental pollution (Bukhari et al., 2020).

**TABLE 2:** Influence of different concentrations of H_3_PO_4_ on the treatment of different feedstocks.

Feedstock	Dry matter (%)	Acid concentration	Temperature (°C)	Time (min)	Saccharification efficiency (%)	References
Corn stover	5	2% H_3_PO_4_	121	120	56	Um et al. (2003)
	12.5	85% H_3_PO_4_–acetone	50	60	67.9	Li et al. (2009)
	8	85% H_3_PO_4_	40	60	48.7	Yu et al. (2019)
	15	84% H_3_PO_4_	50	45	75	Zhang et al. (2007)
Rapeseed straw	12	1% H_3_PO_4_	200	15	93.9	López-Linares et al. (2013)
Sugarcane bagasse	5	0.2% H_3_PO_4_	186	8	56.4	López-Linares et al. (2013)
Sweet sorghum bagasse	12.5	85% H_3_PO_4_	50	30	79	Goshadrou et al. (2011)
Wheat straw	15	1.75% H_3_PO_4_	190	15	86	Nair et al. (2017)
*Achyranthes aspera*	12.5	75% H_3_PO_4_	60	60	86.2	Siripong et al. (2016)
*Sida acuta*	12.5	75% H_3_PO_4_	60	60	82.2	Siripong et al. (2016)

Currently, dilute acid pretreatment is the most feasible approach for industrialization. Various types of equipment reactors have been designed for the technique. According to the kind of usage, there are two types of utilization of dilute acid treatment: a short time for high temperature (180°C) and a long time for low temperature (120°C). Dilute sulfuric acid, nitric acid, hydrochloric acid, phosphoric acid, oxalic acid, maleic acid, formic acid, and acetic acid have been investigated (Mosier et al., 2005). The most widely applied and tested technologies are based on dilute H_2_SO_4_. For example, wheat straw was pretreated under the condition of 1.6% dilute H_2_SO_4_ at 147°C for 30 min, and it was found that the most dramatic improvement of fermentable sugars was the temperature, which was helpful to the hydrolysis of straw biomass (Satari Baboukani et al., 2012). Using dilute H_2_SO_4_ treatment of rice straw, the ratio of recovery glucose was approximately 90% under the best conditions of 1.2% H_2_SO_4_ at 110°C for 14.02 min (Kim et al., 2012).

Dicarboxylic acids could overcome the disadvantages compared with sulfuric acid because they have two pKa values (Kumar and Sharma, 2017). Besides, dicarboxylic acids exhibit excellent performance based on appropriate reaction parameters (temperature and pH values), and straw biomass can be hydrolyzed more efficiently. Oxalic acid and maleic acid are the common dicarboxylic acids employed for the treatment process (Akhtar et al., 2016; Kumar and Sharma, 2017). For example, oxalic acid not only is more environmentally friendly than sulfuric acid but also exhibits fine glycolysis. Furthermore, it produces less side products. Apart from the above-mentioned advantages, maleic acid is beneficial to cellulose degradation to glucose, not glucose hydrolysis (Chen et al., 2017; Kumar and Sharma, 2017). Considering the environmental safety, although acid treatment is the most extensive technique on the industrial scale, less attention has been paid because of its drawbacks and limitations (Mosier et al., 2005; Jeong and Oh, 2011).

Applying HCl-pretreated corn straw could increase the hemicellulose, cellulose, and lignin fractionation, as well as decreasing the activation energy of the reaction process (Chen et al., 2021). Furthermore, a central composite design was used to enhance the sugar recovery efficiency and conversion of cotton and sunflower straw. Various parameters such as the acid concentration, reaction time, and temperature as well as the fermentable sugar yields were optimized, obtaining 20 and 15.5 g L^−1^ fermentable sugars from cotton (121.7°C, 2.28% acid concentration, 36.82 min) and sunflower straw (87.03°C, 3.68% acid concentration, 36.82 min) under optimum treatment parameters, respectively (Yildirim et al., 2021).

Acid treatment has its advantages and disadvantages for the pretreatment of lignocellulosic feedstocks. On the one hand, it could disrupt the lignocellulose and amorphous cellulose. On the other hand, it has high energy consumption of acid recovery and generation of byproducts. In comparison with the concentrated acid, dilute acid pretreatment is more popularly employed, possibly due to the need for lower acid concentration and lesser energy consumption but with higher sugar yields.

## Ionic Liquid Pretreatment

Ionic liquid (IL) is a new type of green solvent and has obtained increasing attention in recent years for the treatment of straw biomass. The IL is composed of organic cations and inorganic anions completely, which exist in liquid form at or below 100°C (Chang et al., 2016). Some unique physicochemical properties are 1) low melting point, vapor pressure, and volatility, 2) low toxicity and hydrophobicity, 3) high stability, polarity, and solubility, 4) high ionic conductivity, 5) less energy cost, 6) simple operation, 7) excellent recyclability, and 8) non-flammable and non-polluting. In addition, the IL is considered a novel salt that could be commonly generated in liquid form at room pressure and ambient temperature. Hence, it is an effective pretreatment method of straw biomass attributed to these unique characteristics (Chang et al., 2016; Kumar and Sharma, 2017). The IL is commonly defined by the term “designer solvents” because its characteristics could be altered and controlled by the choice of cations and anions developed for some particular utilization. Besides, monosaccharides, oligosaccharides, and polysaccharides are soluble in the IL. Despite the IL having numerous distinct features for the pretreatment of straw biomass, there are some drawbacks including high expense, high toxicity, high viscosity, more inhibitors, and the requirement of much energy to recycle the solvent (Kumar and Sharma, 2017).

Currently, numerous straw biomass also is pretreated with many diverse types of ILs. For example, rice straw was pretreated with eight kinds of cholinium amino acid ionic liquids ([Ch][AA] ILs), cholinium lysine ([Ch][Lys]) was reused five times, and the yields of glucose and xylose could reach 80 and 52.2%, respectively. It was shown that the [Ch][AA] ILs could remarkably facilitate the enzyme digestion rate and sugar yield. More importantly, [Ch][Lys] revealed excellent reusability. Therefore, the recycling of ionic liquids has a broad prospect (Hou et al., 2012). Wheat straw was treated under cholinium taurate ([Ch][Tau]) and enzymolysis, and the reducing sugar yields reached 79.7% (Ren et al., 2016). Using 1-ethyl-3-methylimidazolium acetate (EMIMAc) to pretreat rye straw can significantly improve the yield of reducing sugar (Smuga-Kogut et al., 2017).

The presence of water in IL solutions could decrease the recovery cost and viscosity of IL and elevate the utilization rate of biomass, while adding water in the IL can remarkably reduce the process cost. For example, wheat straw was treated with EMIMAc solution containing moisture up to 50%, giving 95% yield of glucose. In addition, bagasse was pretreated with 1-butyl-3-methylimidazolium chloride (BMIMCl) solution with 1.2% HCl, and glucan digestibility reached 94–100%. In the imidazole ionic liquid system containing 20% water, lignin removal and glucose digestibility can be increased by decreasing the pH of the solution (Zhang et al., 2013). EMIMAc containing NaOH was used to pretreat corn stalk, giving 85.69% hemicellulose, 9.1% cellulose, and 87.4% lignin removal under the best conditions of the liquid–solid proportion of 8.7:1 at 98.5°C for 1.31 h (Liu et al., 2018).

Over the past decade, IL pretreatment has been deemed as an effective approach for lignocellulosic biomass fractionation, saccharification, and fermentation (Van Osch et al., 2017; Usmani et al., 2020). In addition, the technology could be used for producing other side products, which could elevate the overall pretreatment economic benefits. However, some key factors could affect the efficiency of straw biomass pretreatment, for instance, the IL nature characteristics (protic or aprotic), treatment conditions (reaction temperature, reaction time, and biomass particle and loading), and different types of biomass such as softwoods and hardwoods and grasses (Halder et al., 2019; Bhatia et al., 2021). Therefore, a variety of ILs would be synthesized to tune their characteristics by adjusting the types of cations and anions. Most importantly, selecting an appropriate IL treatment depends on various lignocellulosic biomass. However, numerous technological and economic challenges exist in the process of IL treatment of straw biomass. For example, the high-cost issue for recovery and reusability of ILs was still the main obstacle in the application of some ILs in industrialization, despite some achievements that have been obtained to design inexpensive ILs (Bhatia et al., 2021). Therefore, the pretreatment of ionic liquids has limited applications.

## Deep Eutectic Solvents

Deep eutectic solvents (DESs) are an effective substitute of ionic liquids in the process of biomass treatment, which are considered one of the most popular types of green solvents for the 21st century. Furthermore, they are quickly emerging and developing according to their variety, design, low cost, green, high tunability, easy-to-synthesize nature, easy recyclability, high solubility, biocompatibility and biodegradability, non-flammability, environmental friendliness, and 100% atom-economic procedures (Paiva et al., 2014; Procentese et al., 2015; Chen and Mu, 2019). The preparation of DESs includes three approaches such as heating and stirring, evaporating technology, and freeze-drying technology (Fernandez et al., 2018). Surprisingly, the presence of DESs can maintain the stability and activity of enzymes. Hence, they have become increasingly popular and attracted attention in numerous fields (Procentese et al., 2015; Chen and Mu, 2019; Rezania et al., 2020).

**FIGURE 5:**
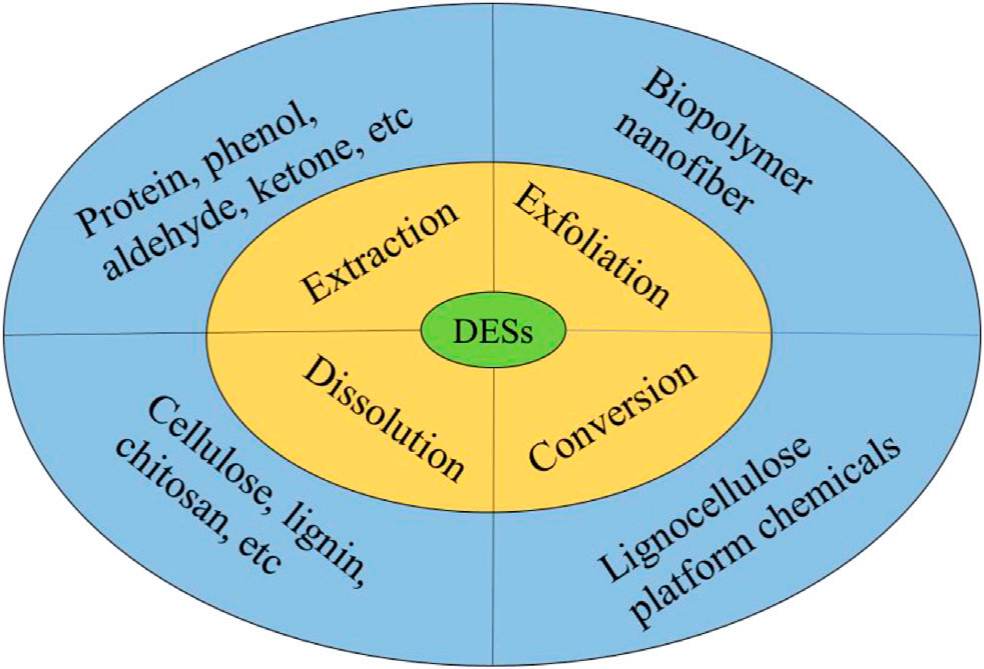
Common DESs are used for biomass treatment and conversion. Adapted with permission from Chen and Mu (2019).

DESs are peculiar compounds containing the components of hydrogen-bonding acceptor (HBA) and hydrogen-bonding donor (HBD), which could be extended into three or more mixtures (Paiva et al., 2014). The intense interaction between the HBD and the HBA not only makes the freezing point or melting point of DESs dramatically lower than those of their mixtures but also disrupts intense hydrogen bonds among straw biomass and improves the efficiency of conversion and the solubility of straw biomass. Common DESs used for biomass treatment and conversion are collected in Figure 5.

**FIGURE 6:**
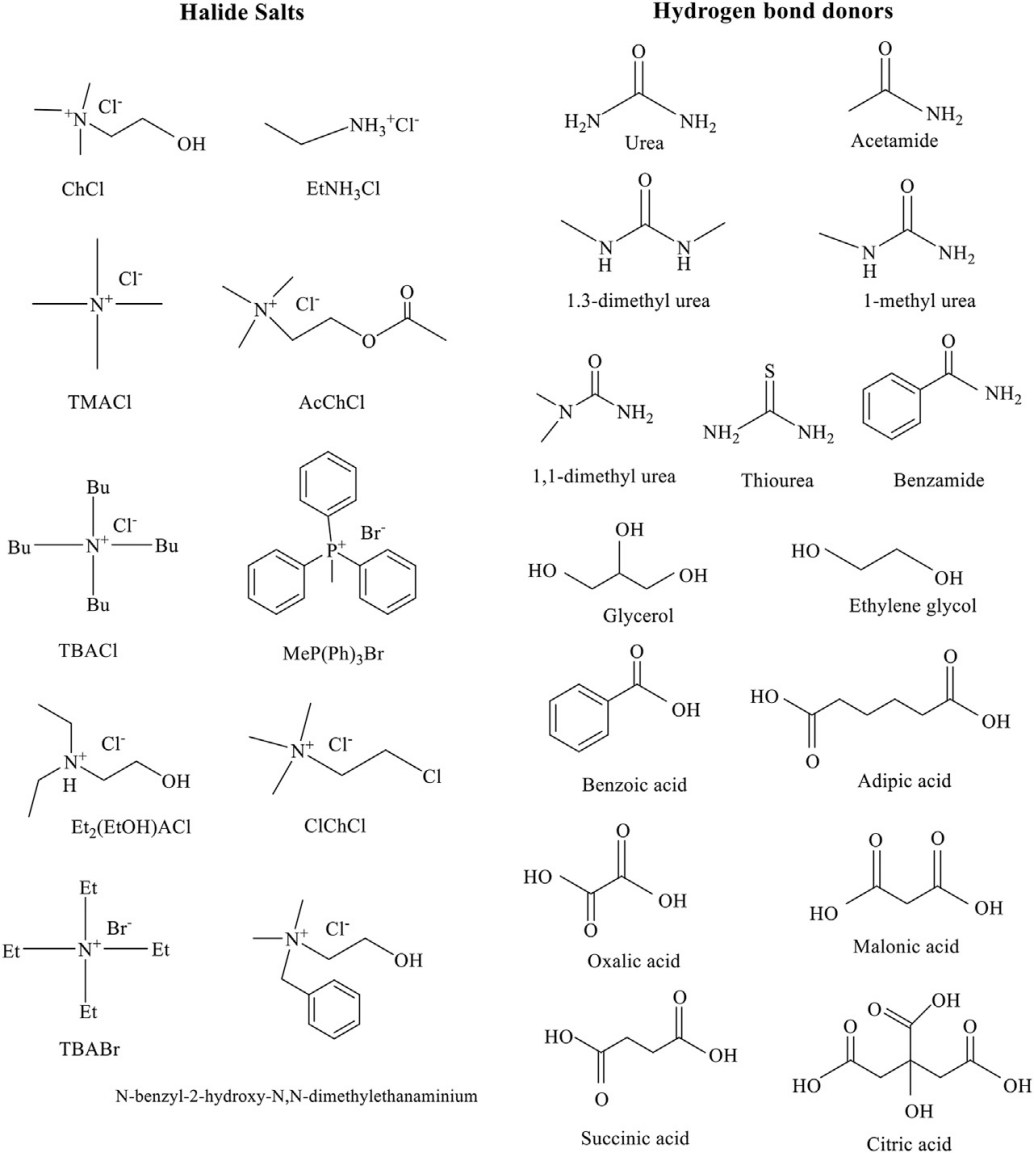
Common structures of hydrogen bond donors and halide salts are utilized in the formation of DESs. Adapted with permission from Zhang et al. (2012).

DESs have mainly been classified into four types according to these general formulas (Table 3) (Smith et al., 2014; Satlewal et al., 2018). Common structures of hydrogen bond donors and halide salts are utilized in the formation of DESs (Figure 6) (Smith et al., 2014). DESs play three roles in the pretreatment process, which can be used as an excellent catalyst, solvent, and substrate. Only understanding the connection with the special structures and properties of DESs can design the ideal and suitable DESs for the pretreatment of various straw biomass (Hou et al., 2017; Ai et al., 2020). Furthermore, using the hydrophobic DESs and water can reduce hygroscopicity and viscosity, respectively (Chen and Mu, 2019; Hooshmand et al., 2020).

**TABLE 3:** General formula for the classification of DESs (Satlewal et al., 2018).

Type	Components	General formula	
I	Metal salt + organic salt	Cat^+^ X^−^ zMClx	M = Zn, Sn, Fe, Al, Ga, In
II	Metal salt hydrate + organic salt	Cat^+^ X^−^ zMClx. yH_2_O	M = Cr, Co, Cu, Ni, Fe
III	HBD + organic salt	Cat^+^ X^−^ zRZ	Z = CONH_2_, COOH, OH
IV	Zinc/aluminum chloride + HBD	MCl_x_ + RZ = MCl_x-1_^+^, RZ + MCl^−^x_+1_	M = Al, Zn & Z = CONH_2_, OH

Notes: Cat^+^, any ammonium, phosphonium, or sulfonium cation; X, a Lewis base, generally a halide anion; Y, a Lewis or Brønsted acid; z, the number of y molecules that interact with the anion.

Some researchers attempted to disclose the mechanism of monocarboxylic acid, dicarboxylic acid, and polyalcohol/ChCl pretreatment utilizing rice straw, and they verified that the acid amount and strength and nature of the HBA play a major role in delignification and enzymatic hydrolysis efficiency of cellulose. For example, ChCl/formic acid treatment of corn stover furnished the excellent glucose yield, removed 66.2% hemicellulose and 23.8% lignin, and significantly improved saccharification efficiency (Hou et al., 2017). After pretreating wheat straw with choline chloride:monoethanolamine (C:M) as the best solvent (70°C, L/S mass ratio of 20:1, 9 h), giving 93.7% cellulose and removing 71.4% lignin, further enzymatic hydrolysis of residue gave the conversion rate of cellulose and xylan of 89.8 and 62.0%, respectively. The results showed that lignin removal, polysaccharide conversion, and reducing sugar yield could be significantly enhanced during pretreatment. Therefore, basic ethanolamine-based DES is a promising solvent for wheat straw treatment, and the obtained efficiency of treatment is better than that of basic ethanolamine DESs and neutral or weakly basic DESs (Zhang et al., 2018).

Numerous choline chloride (ChCl)–based DESs including weakly basic DESs and acidic DESs were commonly utilized to treat straw biomass involving wheat straw, corn stover, corn cob, and rice straw (Zhang et al., 2018). For instance, the pretreatment of corn cob residues with choline chloride and imidazole could achieve 41 g fermentable sugars recovered from 100 g corn cob, accounting for 76% initial carbohydrates (Procentese et al., 2015).

Besides, NADESs are a kind of special DESs, which are naturally occurring ingredients that are individually usually present in food (Vanda et al., 2018). NADESs are defined as mixtures of diverse molar ratios of natural compounds including organic acids and bases, amino acids, sugars, sugar alcohols, choline, urea, and polyalcohol. NADESs are achieved by the synergistic effect between a hydrogen bond acceptor and a hydrogen bond donor. The charge delocalization that occurred is hereafter responsible for decreasing the melting point of the mixture relative to the melting points of the feedstocks (Paiva et al., 2014). The main reason for the phenomenon is the interactions between hydrogen bonds and van der Waals force (Fernandez et al., 2018). According to the chemical properties of their components, NADESs involve derivatives of organic acids, derivatives of choline chloride, mixtures of sugars, and other compounds (Fernandez et al., 2018; Smith et al., 2015). Numerous NADES combinations have been prepared, and the most popular NADESs are choline chloride–based reagents because of the lower cost and easier preparation of high-purity solvents. For instance, rice straw was pretreated by NADESs at the best conditions of the molar ratio of lactic acid/choline chloride of 5:1, the solid loading of 10%, and the enzymatic hydrolysis time of 24 h, giving 333 ± 11 mg g^−1^ reducing sugars and 36.0 ± 3.2% saccharification efficiency (Kumar and Pravakar, 2016). NADES pretreatment of lignocellulosic biomass is more effective than conventional synthetic ionic liquids because it can avoid shortcomings such as poor biodegradability, poor biocompatibility, poor sustainability, high toxicity, and environmental problems (Fernandez et al., 2018). Although a large number of studies on straw biomass treatment with NADES reagents indicated that the major advantage is high-purity lignin obtained, the main shortcoming is the viscosity that is too high to limit the utilization on the treatment of straw biomass (Kumar and Sharma, 2017).

DES pretreatment for the conversion of biomass also shows some disadvantages such as instability at certain reaction conditions, vaporability of DES-like traditional organic solvents, degradation of DESs generating impurities, strong hygroscopicity, high viscosity, probable ecotoxicity, and cytotoxicity. Fortunately, DESs with unique characteristics such as variety and designability would overcome these drawbacks. For instance, a combination of the microwave with DESs can decrease the reaction time and reduce the influence of instability. Using the hydrophobic DESs can reduce hygroscopicity. The presence of water in DESs would reduce the viscosity of some DESs to increase efficiency (Chen and Mu, 2019).

Overall, DESs could selectively dissolve high amounts of lignin while maintaining hemicellulose and cellulose intact as much as possible. Hence, DESs would play an important role in pretreatment techniques of straw biomass, which are proposed as the most promising and environmentally friendly alternatives to traditional solvents for elevating straw biomass conversion. There are plenty of process parameters that could affect the efficiency of DES treatment, for example, 1) properties of lignocellulosic feedstocks including component, crystallinity, and particle size, 2) characteristics of DESs such as the HBD and HBA properties and the molar ratio of HBD and HBA, and 3) reaction conditions of treatment including the effect of the ratio of the solid to the liquid, temperature, and time of DES treatment (Xu et al., 2020; Yoo et al., 2020). Hence, they are of great significance for the development of lignocellulose feedstock conversion, fractionation, saccharification, and fermentation to further research the DES characteristics and reaction conditions. Although many previous studies had reported on DES treatment from various aspects, the exact mechanism of DES interaction with lignocellulose feedstocks still has not been demonstrated (Xu et al., 2020).

## Organosolv Pretreatment

Organosolv treatment is an attractive approach for solubilizing hemicellulose and isolating cellulose as well as extracting almost pure lignin from straw biomass. The common solvents utilized in organic solvent treatment are methanol, ethanol, butanol, glycol, acetic acid, formic acid, propionic acid, acetone, formaldehyde dioxane, glycerin, tetrahydrofuran, phenol, and amines with and without catalyst or mixed with the organic solvent and water. Due to the unique characteristics of organic solvents such as low boiling point, high pressure, easier volatility, and flammability, organosolv treatment is an alternative technology and a promising pretreatment technology. However, expensive investments, high inhibitory products, and not being environmental-friendly are the main disadvantages (Borand and Filliz, 2018).

Different organic solvents and treatment conditions can significantly improve the pretreatment efficiency of organic solvents (Weerasai, et al., 2014). For example, the treatment of organic solvents was reviewed from the aspects of loading amount and particle size of raw materials, the type and concentration of solvents, reaction temperature, time, and pressure (Borand and Filliz, 2018). Furthermore, bagasse was treated with a mixture of ethylene glycol (EG, the boiling point of 197°C) and ethyl carbonate (EC, the boiling point of 260°C) with a high boiling point (EC:EG = 4:1) at 90°C for 30 min with 1.2% sulfuric acid, and 93% glucan enzymatic digestibility was obtained (Zhang et al., 2013).

Glycerol organic solvent pretreatment can remarkably disrupt the complex and recalcitrant structure of straw biomass and selectively remove the partial barrier formed by hemicellulose and lignin to protect cellulose (Sun et al., 2015). Furthermore, atmospheric aqueous glycerol autocatalytic organosolv pretreatment (AAGAOP) can break down the ester bond and glycoside bond between hemicellulose, cellulose, and lignin, improve cellulose accessibility, and promote the conversion and enzymatic digestion of straw biomass (Sun and Chen, 2008). For instance, after wheat straw was pretreated with AAGAOP in the liquid–solid ratio of 20 g^−1^ at 220°C for 3 h, 70% hemicellulose and 65% lignin were removed and 98% cellulose was retained (Sun et al., 2003).

Islam et al. (2021) studied and quantified the benefits of a new technology organosolv (OS)–dilute acid (DA) treatment process for biomass residue conversion. OS–DA treatment obtained about 90% cellulose digestibility from the substrate. Organosolv treatment could dramatically facilitate cellulose digestibility via a one-pot fractionation method from lignocellulosic biomass feedstocks (Zhao et al., 2017). Furthermore, commonly utilized organosolv systems usually use alcohol and water combined with the acid or base catalyst such as water/ethanol and H_2_SO_4_ (Asadi et al., 2017), water/methanol and alkaline (Yuan et al., 2018), and organosolv coupled with a steam explosion (Matsakas et al., 2019).

In general, organosolv treatment is an effective treatment that employs organic solvent at 100–180°C. No exogenous catalyst is required at 185–210°C because the organic acids generated from the lignocellulosic biomass could act as catalysts instead of using organic solvents (Zhao et al., 2017). The advantage of organosolv treatment is that it could obtain the high efficiency of cellulose separation and hemicellulose fractionation, high lignin dissolution, and fewer byproducts’ production, as well as keeping the stability of β-O-4 linkages from avoiding degradation and condensation for downstream applications (Dong et al., 2019). However, there are some disadvantages including solvent recycle and fractionation. Hence, an ideal organosolv treatment should address the problem. Thoresen et al. (2020) summarized how to choose the solvent and improve the component separation as well as the potential for solvent recycle.

## Sulfite Pretreatment

Sulfite pretreatment to overcome recalcitrance of lignocellulose (SPORL) is a novel treatment method for lignocellulosic biomass, which has a strong bioconversion of straw biomass (Wang et al., 2009). Typically, SPORL includes two steps. The first step is biomass pretreated by calcium or magnesium sulfite to degrade hemicellulose and remove cellulose at pretreatment conditions of 160–180°C for a short time and pH value of 2–4. The second step applying a disc refiner–treated biomass can dramatically decrease the size of biomass (Kumar and Sharma, 2017).

SPORL has plenty of advantages that are as follows: dramatic cellulose decomposition, obvious hemicellulose removal, and excellent hydrolysis efficiency. Besides, it can save energy consumption, increase enzyme loading, and enhance the fermentation process (Wang et al., 2009). However, there are some drawbacks, such as sugar degradation and high cost, requiring to be addressed. For instance, SPORL can achieve almost 100% conversion of cellulose; the optimal conditions would be 180°C, 30 min, 4% sodium bisulfite charge, pH value of 2.0–4.5, and enzymatic hydrolysis time of 10 h. Currently, Na_2_S, Na_2_SO_3_, Na_2_CO_3_, and NaOH are utilized in SPORL of diverse straw biomass (Yang et al., 2013).

Ammonium sulfite treatment is a promising treatment technology to achieve fermentable sugars from lignocellulosic feedstocks. For example, wheat straw was pretreated by 20% (w/w) ammonium sulfite assisted with 4% Na_2_CO_3_ at 180°C for 1 h, obtaining 99.9% glucan yield and 88.0% xylan yield (Qi et al., 2018). In addition, various lignocellulosic biomass including switchgrass, lodgepole pine, poplar, and red pine was pretreated with SPORL, hemicellulose was removed, a part of cellulose was degraded, and lignin was sulfonated, finally generating a hydrophilic polyphenolic structure (Silveria et al., 2015).

## Oxidative Pretreatment

Oxidative treatment of lignocellulosic biomass involves H_2_O_2_, peracetic acid, ozone, oxygen, or air, and many chemical reactions involving electrophilic substitution, side-chain displacements, and oxidative cleavage of aromatic ring ether linkages may take place during oxidative pretreatment. The acids and inhibitory compounds are generated from lignin fragmentation and oxidization, which could influence the yield of fermentable sugar and the efficiency of enzymatic digestion (Kumar and Sharma, 2017).

Oxidation treatment involves ozonolysis, wet oxidation, and photocatalysis (Chen et al., 2017). Ozone has high oxidability that could degrade the lignin and decompose the hemicellulose and usually dispose of various straw biomass. Using ozone treatment of straw biomass in an ambient environment could dramatically promote the removal of lignin without generating any inhibitors (Talebnia et al., 2010). Unfortunately, the process of ozone treatment required a lot of ozone, which is uneconomical, unfeasible, energy-consuming, and of high cost (Chen et al., 2017).

Wet oxidation is a suitable process of disposing straw biomass with water and air/oxygen at stringent temperature, pressure, and time. The method can cleave the lignin, solubilize the hemicellulose, promote the susceptibility of cellulose, and decrease the generation of byproducts. The technology needs a higher temperature (above 120°C) and higher pressure (0.8–3.3 MPa) but could obtain an excellent pretreatment efficiency compared with other treatment approaches (Talebnia et al., 2010; Chen et al., 2017). The major shortcomings of the technique are the requirement of high temperature and pressure, high expenses, oxidizing agents, and unique equipment (Xu et al., 2020; Das et al., 2021). Furthermore, another major drawback of the treatment is that it has low efficiency for generating fermentable sugars, attributed to degrade a large amount of hemicellulose (Lucas et al., 2012; Machineni, 2020).

A combination of H_2_O_2_ and peracetic acid with alkaline (NaOH) could significantly enhance the reducing sugar yields and the enzymatic hydrolysis efficiency as compared to the NaOH treatment alone. For example, when pretreatment of wheat straw using 1% H_2_O_2_ and alkaline was performed at 25°C for 18–24 h and a pH value of 11.5, 50% lignin was removed and most of the hemicellulose was dissolved, which was more efficient than only NaOH treatment. Furthermore, the enzymatic digestion efficiency was improved, giving almost 100% conversion when the value of pH reached 11.5 (Talebnia et al., 2010).

Novel oxidative treatment technology of straw biomass fractionation has been reported, which applies both O_2_ and H_2_O_2_ as co-oxidants under alkaline conditions to simultaneously elevate the efficiency of straw biomass being converted into fermentable sugars and enhance the purity and stability of lignin (Yuan et al., 2021).

## Steam Explosion Pretreatment

Steam explosion (SE) pretreatment is a common, co-effective, and promising technology, which is utilized in industrial conditions for the treatment of straw biomass (Agudelo et al., 2016; Dai et al., 2020). SE is usually initiated at high temperature (160–260°C) and pressure (0.69–4.83 MPa) for seconds or several minutes, and then the pressure is suddenly released. Steam quickly penetrates lignocellulose and then undergoes sudden decompression, and water is evaporated fast forming an explosion inside the fibers. SE is a high-energy and high-pressure consumption treatment technology due to its requirement for high temperature and pressure (Smichi et al., 2020). Acetic acid could be generated from acetyl groups of hemicellulose by autohydrolysis at high temperatures. Hence, water is regarded as an acid in the treatment process. The yield of fermentable sugars can be enhanced because hemicellulose is dissolved, lignin is removed, and cellulose hydrolysis is intensified in the whole process (Oliveira et al., 2013; Vochozka et al., 2016). Some key parameters affect the efficiency of SE pretreatment such as steam temperature, moisture content, and particle size of feedstocks as well as reaction time. The addition of H_2_SO_4_ or SO_2_ in SE has been considered the best manner to increase fermentation efficiency (Talebnia et al., 2010; Vochozka et al., 2016).

In comparison with other treatment technologies, SE treatment could be applied to treat straw biomass to dramatically not only reduce the requirement of hazardous chemicals and reaction time consumption but also facilitate the fractionation of lignin and achieve high purity, high quality, and high stability of lignin. Various lignocellulose biomass such as rice straw, corn stover, wheat straw, sugarcane bagasse, and sunflower stalks has been pretreated by SE treatment to achieve fermentable sugars (Smichi et al., 2020). For instance, rice straw exhibited an effective enhancement in physicochemical characteristics as compared to the unpretreated counterpart. 53.46% cellulose digestion and 49.54% hemicellulose degradation were achieved, which were increased by 13.72 and 16.79% as compared to the untreated counterpart, respectively (Zhou et al., 2016). Besides, rice straw and corn stalk treated with SE can dramatically enhance the strength of internal bonding and water tolerance, degrade hemicellulose, and transform lignin (Kurokochi and Sato, 2020).

In addition, a combination of SE and fungal treatment of corn stalk achieved 313.31 g kg^−1^ glucose yield under the optimum conditions of 1.7 MPa using SE assisted with *Phellinus baumii* for 21 days, which is 2.88 and 1.32 times higher as compared with that of an untreated corn stalk and 1.7 MPa SE, respectively (Li and Chen, 2014). Furthermore, sugarcane bagasse was employed by SE treatment coupled with acetosolv at 168°C for 10 min, and the lignin yield increased by about 17% in comparison with the untreated one (Marques et al., 2021).

SE showed excellent pretreatment performance compared to some other treatment methods. This involves the potential for the remarkable enhancement in enzyme hydrolysis, which is more environmental-friendly and economic, with less hazardous reaction conditions and higher sugar recovery. In addition, SE can employ the larger chip size and does not need the acid catalyst as well as its feasibility in the industry. However, SE treatment has some shortcomings such as high-energy input and fermentation inhibitors (e.g., formic acid, 5-hydroxymethylfurfural, acetic acid, and furfural), which would reduce the fermentation efficiency (Smichi et al., 2020; Balan et al., 2020).

## Supercritical Fluid Pretreatment

Supercritical fluids (SCF) are substances that above their critical points of temperature and pressure, and the fluids don’t present vapor-liquid phase transition (Akhtar et al., 2016; Capolupo and Faraco, 2016). A fluid is called supercritical when it is above the critical temperature (T_c_) and pressure (P_c_) and below the pressure required for condensation. When the gas and liquid phases at supercritical conditions coexist, they would not be distinguished and reach the critical point (Morais et al., 2015). In the supercritical area, the unique liquid acts as a special solvent due to its unique characteristics such as gas-like viscosity, diffusion rate, and intermediate between gas and liquid, which could enhance its penetrability toward straw biomass. The SCF could penetrate the small pores of biopolymers (e.g., cellulose), which is useful to solve the problem of mass transfer encountered in other treatment technologies and further facilitate the saccharification and fermentation efficiency (Akhtar et al., 2016). The solubility of substrates could easily be increased when near to the critical point, because it could be altered by both the system temperature and pressure (Morais et al., 2015). Classical treatment technologies usually require severe conditions such as high temperature, high pressure, and many side products in the reaction process. In sharp contrast, the SCF approach could operate under mild treatment conditions to achieve high fermentable sugars and low inhibitors (Ponnusamy et al., 2019).

**FIGURE 7:**
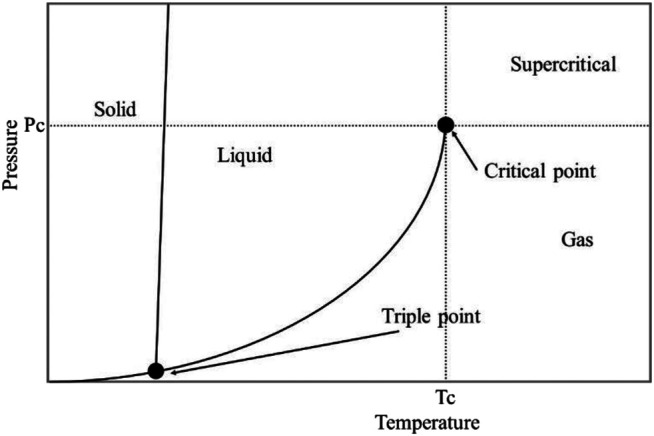
Pressure–temperature phase diagram of a compressible fluid with solid–liquid–gas phase and supercritical region. P_c_: critical pressure; T_c_: critical temperature. Reproduced with permission from Li et al. (2020).

Carbon dioxide, ammonia, water, and hydrocarbons (propane and butane) are the most common supercritical fluids. Supercritical carbon dioxide (Sc-CO_2_) is one of the most popular utilized compressed fluids for lignocellulose feedstock processing attributed to generate no byproducts without requiring any separation process (Escobar et al., 2020). Moreover, Sc-CO_2_ is based on the application of CO_2_ as a supercritical fluid. The fluid exhibits “gas-like” mass transfer characteristics and “liquid-like” solvating power (Li et al., 2020; Ramezani et al., 2020). Besides, CO_2_ is non-flammable and non-toxic, which could be adjusted to supply a higher diffusion co-efficiency, strong solubility, excellent diffusivity, and strong recoverability (Rezakazemi et al., 2017; Rezakazemi et al., 2019). CO_2_ has its properties such as low temperature (31°C) and pressure (7.4 MPa), and formation of the supercritical CO_2_ region, with liquid-like density and gas-like viscosity/diffusivity (Figure 7), which could elevate the permeability of the small pores of feedstocks, further breaking down the conjunctions between hemicellulose and cellulose as well as decreasing its crystallinity (Carneiro et al., 2016; Soroush et al., 2019).

Using Sc-CO_2_ pretreatment of biomass exhibits some benefits such as lower cost of CO_2_, moderate pressure, lower temperature, and higher solid loadings. Importantly, CO_2_ is more easily available and transported. In addition, when using the Sc-CO_2_ treatment of straw biomass, CO_2_ generates carbonic acid and enhances the enzymatic digestion efficiency (Rezakazemi et al., 2017; Rezakazemi and DarabiSoroushMesbah, 2019). Moreover, CO_2_ can penetrate the small pores of hemicellulose, cellulose, and lignin, leading to disruption of hemicellulose and cellulose structures, increment of cellulose accessibility and enzymatic hydrolysis, and minimization of inhibitors. However, the high requirement of reactor equipment limited the extensive application (Kumar and Sharma, 2017; Li et al., 2020).

Sc-CO_2_ treatment has been applied for lignocellulosic biomass, which can significantly improve the fermentable sugar yields (Gao et al., 2010). For instance, corn stover pretreated with Sc-CO_2_ could remarkably enhance the glucose yield when increasing the temperature and pressure. Under the best conditions of 75% water content of corn stover at 150°C and 24.1 MPa for 1 h, 30% glucose yield was achieved, which was increased by 18% than the unpretreated one (Narayanaswamy et al., 2011). Moreover, the Sc-CO_2_ treatment of rice straw could achieve 32.4 ± 0.5% yield of glucose at 110°C and 30 MPa for 30 min, while the untreated rice straw only achieved 27.7 ± 0.5% yield of glucose. Furthermore, sugarcane bagasse was also treated by Sc-CO_2_ at 40°C and 10 MPa after 120 min, giving 60% fermentable sugars (Li et al., 2020).

In addition, Sc-CO_2_ treatment on rice straw and wheat straw and subsequent enzyme hydrolysis could achieve 100 and 32% yield of glucose, respectively (Akhtar et al., 2016). Another research showed milling of the corn cob and corn stalk with a water content of 50% into particles of 0.39–0.83 mm followed by pretreatment with Sc-CO_2_ and an Sc-CO_2_/ultrasound combination. Low and high levels of pretreatment temperature (120–170°C), process time (0.5–4 h), and pressure (15–25 MPa) were selected. The total reducing sugar yields of the corn cob and corn stalk were enhanced by 50 and 29.8% under Sc-CO_2_ treatment alone, while the total reducing sugar yields were increased by 75 and 13.4% under combined pretreatment, respectively (Yin et al., 2014). Overall, the reaction temperature, pretreatment duration, water content, residence pressure, and biomass loading affect the efficiency of Sc-CO_2_ treatment.

Besides, to facilitate the pretreatment efficiency such as fermentable sugar yield and lignin fractionation, a small part of co-solvent (ethanol) could be assisted with Sc-CO_2_ (Ramezani et al., 2020). The water present in the straw feedstocks together with CO_2_ under the critical conditions, and the acidity of *in situ* generated carbonic acid is dramatically beneficial to biomass fractionation, saccharification, and fermentation. Hence, Sc-CO_2_ treatment is a highly promising approach for straw biomass conversion, particularly when combined with the up-to-date or cutting-edge biorefinery (Li et al., 2020; Ramezani et al., 2020).

Other supercritical fluid approaches such as supercritical ammonia and supercritical ethanol are also employed in the area of biomass fractionation and conversion. In addition, in comparison with subcritical ethanol, the supercritical ethanol pretreatment exhibited higher lignin fractionation and higher purity and stability of cellulose (Escobar et al., 2020). Then, the water present in lignocellulosic feedstocks coupled with carbon dioxide produces carbonic acid, which could facilitate the efficiency of hemicellulose degradation (Daza Serna et al., 2016; Fockink et al., 2018).

## SO_2_ Explosion Pretreatment

SO_2_ explosion treatment technology is analogous to carbon dioxide explosion pretreatment, which could catalyze the solubilization of hemicellulose by adding an external acid, promote cellulose hydrolysis, and reduce the pretreatment temperature (Chen et al., 2017). Additionally, SO_2_ dissolved in the water could generate the medium–strong H_2_SO_4_, thus producing H^+^ to promote the glucose generation from cellulose hydrolysis, in which the cellulose hydrogen bonds are disrupted by the medium H_2_SO_4_ (Hassan et al., 2018). Furthermore, SO_2_ in water could transform hemicellulose into many fermentable sugars while generating low inhibitory products. Moreover, SO_2_ can be dissolved into water and extracted from water by steam stripping. Therefore, SO_2_ also can be reused (Liu et al., 2012). Furthermore, SO_2_ also could enhance the efficiency of lignin fractionation (Silveria et al., 2015). However, there are some disadvantages of pretreatment such as the high demands of reactor equipment and numerous degradation products in the case of using acids (Chen et al., 2017).

The SO_2_-catalyzed steam explosion has been utilized in various lignocellulosic materials. For example, sugarcane bagasse was pretreated by SO_2_-catalyzed steam explosion followed by enzyme digestion, and 57% pentose was achieved under the optimum conditions of 2% moisture content of SO_2_ as a catalyst at 190°C for 5 min. After further enzymatic hydrolysis, 87% total sugars and 60% xylose conversion were obtained at 2% water-insoluble solid contents, respectively. More importantly, there were almost no inhibitors generated in the reaction process (Carrasco et al., 2015). In addition, corn straw was pretreated with an explosion approach at 190°C with 3% SO_2_ for 5 min, achieving 74% yield of xylose (Chen et al., 2017).

## Ammonia Fiber Expansion

Ammonia fiber expansion (AFEX) is considered a thermochemical treatment technology, which applies anhydrous ammonia or lower moisture at correspondingly moderate temperatures (60–120°C) and pressures (1.72 MPa) for a short time (5–30 min) and rapidly releases the pressure. The process of treatment bears a resemblance to SE with only liquid ammonia replacing water. This technology dramatically elevated cellulose accessibility and the enzyme hydrolysis efficiency because the crystallinity and polymerization were obviously increased, hemicellulose was effectively hydrolyzed, a large part of lignin was depolymerized and removed, and the size and number of micropores were enhanced in the cell wall (Karp et al., 2013; Rabemanolontsoa and Saka, 2016).

In addition, ammonia is regarded as a good catalyst due to the enhancement of the accessible surface area, the reduction of inhibitors, high lignin removal, moderate reactions, and low cost (Balan et al., 2009; Baral et al., 2014). Furthermore, ammonia has high volatility to increase the efficiency of recovery and reuse. The most important is that the rest of the ammonia in the residue biomass could be deemed the excellent nitrogen resources applied for further fermentation steps and with no need for the washing step to the benefit of hydrolyzing the high solid loading (Krishnan et al., 2010). The loadings of ammonia and water, reaction temperature and time, treatment times, and pressure were the main factors for the AFEX pretreatment (Rabemanolontsoa and Saka, 2016).

AFEX treatment can break down the biomass recalcitrance and elevate the hydrolysis activities and conversion efficiency of different feedstocks, which has been utilized in various lignocellulosic materials including wheat straw, rice straw, corn cob/stover, and sugarcane bagasse (Lau et al., 2010). It was reported that AFEX treatment of corn stover under the best reaction conditions could achieve approximately 98% theoretical glucose yield (Balan et al., 2009). In addition, *Miscanthus* was pretreated by AFEX under the desirable conditions involving ammonia-to-biomass loading of 2:1 (w/w), reaction temperature of 160°C, water content of 233%, reaction time of 5 min, and enzymatic hydrolysis time of 168 h (Murnen et al., 2008). Besides, AFEX treatment of wheat straw could be carried out with aqueous ammonia (25% w/v) in replacement of liquid ammonia as well, giving 90% conversion rates (Kamm et al., 2017). Furthermore, AFEX was used for the pretreatment of rice straw, and the sugar loss was less than 3% (Zhong et al., 2009). Moreover, sugarcane bagasse pretreated by AFEX treatment could decompose the ester linkages and complex bonds of lignin and significantly increase the solubilization of hemicellulose and transformation of cellulose. As a result, 85% glucan and 95–98% xylan were obtained from cellulose and hemicellulose during enzymatic hydrolysis, respectively (Susan et al., 2013).

AFEX treatment technology is similar to the SE approach. The straw biomass is swollen by AFEX, which could elevate the surface area and disrupt the linkage of lignin–carbohydrate (Behera et al., 2014; Mathew et al., 2016; Kumar and Sharma, 2017). For example, AFEX coupled with dilute acid treatment of corn stover displayed the dramatic enzymatic digestion attributed to the excellent effect of AFEX. However, AFEX pretreatment is still difficult for commercial-scale utilizations due to the problem of ammonia recovery (Rezania et al., 2020; Yoo et al., 2020).

## Physical Treatment

### Microwave Pretreatment

Microwave treatment can selectively transfer energy to various materials, which has been usually utilized for the pretreatment of straw biomass (Hassan et al., 2018). The microwave interacts with polar molecules and leads to rapid and volumetric heating (Isci et al., 2020). In the process of microwave irradiation, on the one hand, the direct interaction of specimen ion conduction or dipole rotation produces heat energy. On the other hand, dipole rotation disintegrates the biomass structure into simpler molecules, leading to the molecular collision. The heat energy produced by molecular collision is helpful to the expansion of fibers, resulting in crushing biomass, which facilitates the subsequent hydrolysis efficiency (Tsegaye et al., 2019; Tan et al., 2020).

Microwave treatment has been demonstrated to be a promising technology attributed to its obvious advantages including shorter reaction time, faster heat transfer, better selectivity, uniform volume heating, simple operation, lower energy cost, and lower generation of byproducts (Kumar and Sharma, 2017; Hassan et al., 2018). However, fermentable sugar production from straw biomass is accompanied by numerous shortcomings such as expensive cost, high requirement of equipment, non-thermal influence, specific reactors, and the compatible treatment process (Gong et al., 2010; Li et al., 2016).

Generally, microwave-assisted other pretreatment processes (e.g., alkali, acid, and salt) also showed significant effects. For example, wheat straw was pretreated at 160°C with 1.5% NaOH and 15 min microwave irradiation, a lot of lignin was removed, and the high content of cellulose was retained, which increased the reducing sugar yield (Tsegaye et al., 2019). In addition, with coupling of microwave treatment and acetic and propionic acid treatment of rice straw, lignin removal was 46.1 and 51.54%, while the sugar yield was 71.41 and 80.08%, respectively. It was found that the most important impact factor was the strength of the microwave, the second one was the proportion of solid–liquid, the third one was the concentration of acid, and the final one was the time of irradiation (Gong et al., 2010; Elsayed et al., 2018).

The main advantage of microwave pretreatment is that it can dramatically shorten the reaction time with the assistance of the microwave. Therefore, it is necessary to combine the microwave with other technologies in the pretreatment of straw biomass. However, the rigorous requirement of equipment is still needed, attributed to the high temperature.

## Electron Beam Irradiation

A linear electron accelerator produces electron beam ionizing radiation, which is regarded as the physical pretreatment process. Electron beam irradiations (EBIs) could disrupt the structure of cellulose, hemicellulose, and lignin, decrease the polymerization degree, and increase the hydrolysis efficiency (Hassan et al., 2018). There are some advantages of EBI pretreatment such as higher selectivity, no requirement for toxic chemicals, shorter treatment time, and more eco-friendly and easier control (Grabowski, 2015). For example, it was reported that the yield of reducing sugar of bagasse was enhanced by three times as compared to the untreated one under the EBI dose of 400 kGy (Jiao et al., 2020). Besides, using EBI treatment of rice straw, 52.1% glucose of theoretical maximum was obtained, while the untreated one only achieved 22.6%. The absorbed dose was 80 kGy, and the accelerating voltage was 1 MeV with a beam current of 0.12 mA (Bak et al., 2009). In addition, rice straw pretreated by soaking-based EBI has higher efficiency than the commercial EBI. It is considered an environmental-friendly pretreatment, which not only does not generate inhibitors but also markedly improves the yields of fermentable sugars and enhances enzymatic hydrolysis (Bak, 2014).

However, EBI pretreatment has often manifested more effective results for the increment of glucose yield through the combination of other methods (Kim et al., 2014; Leskinen et al., 2017; Xiang et al., 2017). For instance, in combination of EBI with alkali pretreatment of rice straw, the content of the cellulose was increased by 31.6%, while the content of lignin was decreased by 13.1%. The sugar yield of the treated one was enhanced with the increase in the dose of irradiation (Kim et al., 2014).

High-energy electron radiation pretreatment is one kind of EBI, which is already used in lignocellulose pretreatment. Lignocellulosic biomass is exposed to high-energy electron radiation and could achieve high efficiency of biomass conversion (Nalvaiko et al., 2015; Zhang et al., 2020). High-energy electron radiation treatment is an appropriate approach for the mass generation with low cost and high efficiency in comparison with other methods. Most importantly, irradiation dosage is easy to control, which is the only key factor for the efficiency of pretreatment (Fernandes et al., 2015).

High-energy electron radiation could successfully enhance the conversion efficiency and enzyme digestion rate via launching radiation to the feedstocks. The advantages of this technology include 1) reducing cellulose polymerization, 2) improving the moisture content, 3) disrupting the cellulose structure, 4) increasing cellulose accessibility, and 5) decreasing environmental pollution. However, high cellulose loss, expensive cost, and difficulty in large-scale industrial production are the main drawbacks (Mosier et al., 2005; Hassan et al., 2018).

The higher radiation led to the lower yield of sugars because it would induce the ring of glucose and oligosaccharide degradation (Chen et al., 2017). The structure of polymeride and the dosage of radiation can influence the reaction induced by electron radiation. High-energy electron treatment has been applied to various straw biomass involving rice straw, bagasse, corn stalk, rice husk, wheat straw, and peanut husk (Yang et al., 2008). Moreover, the combination of radiation with chemical technologies can significantly improve the degradation amount and delignification yield, particularly the conjunction of radiation and alkali involving sodium hydroxide.

## Ultrasonic Pretreatment

**FIGURE 8:**
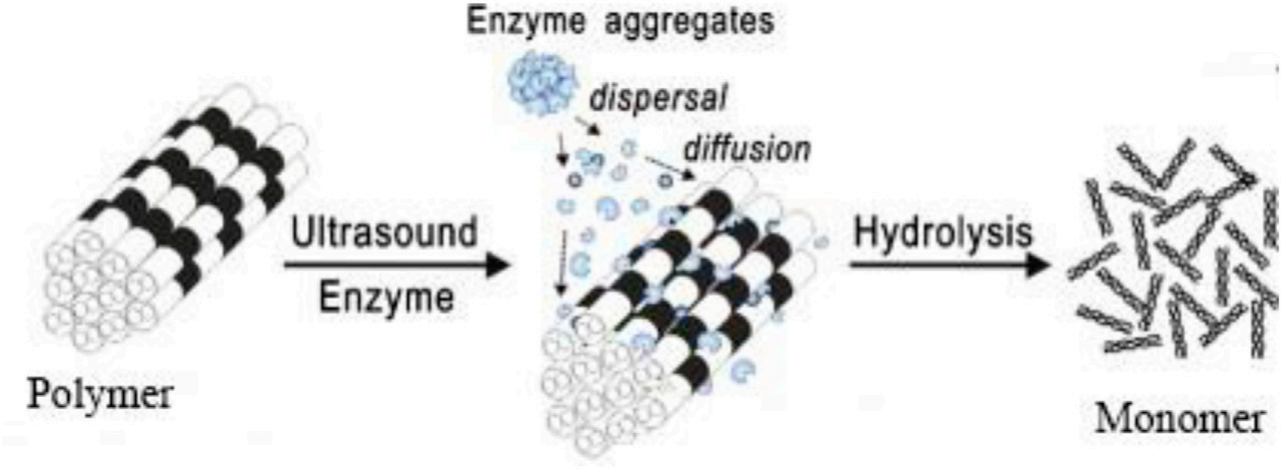
Mechanism of prevention of enzyme agglomeration and depolymerization of lignocellulose using sonication. Reproduced with permission from Gogate (2013).

Ultrasound effects consist of mechanoacoustic and sonochemical effects. Ultrasound treatment can produce high temperature and pressure in the localized, generate highly active free radicals, change the structure of the surface, and enhance the permeation of solvents and heat into cells to facilitate mass transfer. Some researchers reported the mechanism of enzymatic hydrolysis such as agglomeration and depolymerization of lignocellulose under ultrasonic pretreatment (Figure 8) (Gogate, 2013). Moreover, ultrasound pretreatment can cleave the α-O-4 and β-O-4 linkages, and some small cavitation bubbles were formed due to the cleavage of polysaccharide and components of lignin (Dinh Vu et al., 2017; Hassan et al., 2018). Because many small cavitation bubbles were formed by ultrasound treatment, which disrupted the cellulose and hemicellulose fractions, reduced pretreatment time and enzyme consumption improved the hydrolysis process effectively and increased the accessibility of cellulose-degrading enzymes and the yield of reducing sugars (Gogate, 2013; Akhtar et al., 2016; Kumar and Sharma, 2017). Furthermore, the maximum influence of cavitation is generated at 50°C, which is also a suitable temperature for numerous cellulose-degrading enzymes.

Numerous parameters including frequency, power consumption, solvent type, dissolved gas type, reactor geometry, and stirring could influence the efficiency of ultrasound (Silveira et al., 2015). Therefore, ultrasound requires to conjunct with other methods for pretreatment of straw biomass to enhance the efficiency of delignification and enzyme saccharification (Xi et al., 2013; Dinh Vu et al., 2017). For instance, the sugar content of bagasse was 43.9 g/L under dilute acid hydrolysis. The concentration of total sugar was increased by 29.5% (Xi et al., 2013). Moreover, when corn stalk was pretreated by combining ultrasonic with ammonium bicarbonate under the optimized conditions including the reaction temperature of 42°C, the reaction time of 11 min, and the liquid–solid proportion of 12:1, the saccharification ratio was 82.61%, which was dramatically enhanced by 355%, compared with CK (Jiao et al., 2020).

At present, numerous previous investigations reported low-frequency ultrasound treatment, while the high-frequency one requires further exploration for lignocellulosic biomass pretreatment. Another study also reinforced the excellent potential of ultrasound treatment for converting lignocellulosic feedstocks into fermentable sugars (Chatel, 2016). Furthermore, ultrasound treatment coupled with green approaches (microwave treatment) and green solvents (SCFs, ILs, DESs, biocatalysts) would display superior effectiveness to the single pretreatment approach (Silveira et al., 2015; Chatel, 2016; Borand and Filliz, 2018).

## Pyrolysis Pretreatment

Thermochemical treatment involves pyrolysis and gasification according to the operating temperatures. Pyrolysis utilized for the treatment of straw biomass with a temperature above 300°C can disintegrate cellulose into hydrogen, carbon monoxide, and residual char. Pyrolysis treatment could disrupt the cellulose structure of biomass, enhance the calorific value and hydrophobicity, and increase the stability of biomass (Talebnia et al., 2010; Gogate, 2013). Moreover, oxygen is helpful to the pyrolysis process (Akhtar et al., 2016; Chen et al., 2017). In addition, pyrolysis treatment is an endothermic process where less input of energy is achieved, which has many advantages of straw biomass pretreatment (Akhtar et al., 2016; Yuan et al., 2018; Tan et al., 2020).

Of the straw biomass pretreatment processes and biorefinery processes, pyrolysis treatment has been deemed as a common technology for straw biomass disposal, which is more popular. The type of pyrolysis, reaction conditions, and biomass material features can influence the distribution of products and the yield of every end product (Kumar and Sharma, 2017). For example, pyrolysis pretreatment of nutshells altered the physical and chemical characteristics under various temperatures. Furthermore, the mild acid (H_2_SO_4_) leaching of the pretreatment process furnished 80–85% conversion of cellulose at 97°C for 2.5 h (Akhtar et al., 2016).

Lignocellulosic feedstocks consist of a certain part of ash that would dramatically influence the efficiency of pyrolysis treatment (Wang et al., 2017). The major components of ash are alkali and alkaline earth metals (AAEMs) such as K, Ca, Na, and Mg, which could affect the biomass conversion efficiency and pyrolysis behaviors as well as mass balance (Haddad et al., 2017). Recent researches reported that it is necessary to remove or passivate the AAEMs of biomass for enhancing the separation efficiency from lignocellulose pyrolysis (Zhou et al., 2021). Applying acid leaching treatment such as diluted H_2_SO_4_, HCl, and dilute acetic acid solution could obviously remove ash from lignocellulosic materials (Lorenci Woiciechowski et al., 2020; Xin et al., 2020). For example, corn cob was pretreated with FeSO_4_–H_2_O_2_ solution under acid condition, obtaining the highest levoglucosan production enhanced by 95% in comparison with the untreated one because of high-efficiency AAEM removal (Wu et al., 2020). Therefore, to obtain the optimum fermentable sugars, a special acid infusion could be added into lignocellulose before pyrolysis (Zhou et al., 2021).

Furthermore, the efficiency of pyrolysis treatment could be elevated during the presence of O_2_ at lower temperatures. Moreover, 85% monomeric sugars were obtained from cellulose under pyrolysis treatment assisted with mild H_2_SO_4_ from agricultural wastes (Das et al., 2021). In addition, wheat straw was pretreated by pyrolysis combined with *Pleurotus ostreatus* in a fixed-bed reactor, showing high delignification and fermentable sugars (Zhang et al., 2021). Hence, selective fungal pretreatment on lignocellulosic feedstocks could improve the efficiency of delignification and cellulose enrichment as well as the yield of fermentable sugars.

In the biorefinery process, bio-oil is usually produced from lignocellulose feedstocks in the pretreatment of pyrolysis. In contrast, fermentable sugar formation from straw biomass by the pyrolysis treatment technology studies is limited. The type of pyrolysis, reaction conditions, and biomass material features can influence the distribution of products and the yield of every end product.

## Mechanical Comminution

The mechanical comminution approach includes extrusion and milling, which is deemed the most traditional technology treatment and can markedly modify the particle size of straw biomass (Kumar and Sharma, 2017). The total hydrolysis production could be enhanced by 5–25% via reducing the particle size of straw to reduce cellulose crystallinity and polymerization degree, enhancing the specific surface area, and further facilitating the process of saccharification and fermentation. The mechanical comminution approach could make the pretreatment process easier and more efficient, whereas it is usually utilized in combination with other treatment technologies because it does not alone satisfy the efficient treatment of raw materials with three components’ contents (Wang et al., 2018).

Extrusion pretreatment is a continuous process and has many advantages such as shorter reaction time, lower cost, higher solid loadings, easier control, moderate conditions, unformed inhibitors, unrequired additives, and environmental friendliness (Gu et al., 2018; Wang et al., 2020). A twin-screw extrusion treatment of corn straw, 45 g/L glucose, and 40 g/L xylose were obtained after enzyme digestion by altering the distribution of particle size and the space structure (Jiao et al., 2020).

Mechanical grinding (milling) involves chipping and grinding procedures. Milling treatment approaches may be involved in dry milling and wet milling by applying either ball or disk grinding elements. Wet milling exhibited higher efficiency than dry milling of straw biomass. The grinding element number and size and substrate particle size are the most important influence factors (Silveria et al., 2015). For example, rice straw was pretreated by wet and dry milling, under the treatment conditions such as the particle size of 0.5 mm and ball speed of 350 rpm/min ground for 30 min (Kumar and Sharma, 2017). Chipping could decrease the size of straw to 10–30 mm, while grinding could decrease to 0.2 mm, which exhibited the best enzymatic efficiency and highest yield of sugars (Kumar and Sharma, 2017; Liu et al., 2018).

Mechanical comminution pretreatment is usually applied before other technologies to make the components of lignocellulosic materials easier to be separated, which is beneficial to the subsequent treatment. Some researchers manifested that the mechanical pretreatment had a remarkable effect on removing lignin and hemicellulose from various straw biomass. Moreover, when coupled with other treatment techniques, mechanical pretreatment exhibits better performance and can further increase the fermentable sugar yields. For instance, applying alkaline (e.g., NaOH, Ca(OH)_2_, KOH, and NH_3_·H_2_O), mineral (e.g., H_3_PO_4_, HCl, H_2_SO_4_, H_2_SO_4_, and HNO_3_), and organic (e.g., CH_3_COOH and HCOOH) acids combined with mechanical pretreatment could dramatically improve fermentable sugar yields due to their high delignification efficiency (Lin et al., 2010; Silveria et al., 2015).

## Biological Pretreatment

The abovementioned treatment techniques need costly equipment, much energy consumption, and the application of harmful chemicals leading to environmental pollution. In sharp contrast, biological treatment can overcome these drawbacks, which has attracted increasing attention possibly due to high delignification efficiency, low cost, and the absence of the pollution pretreatment process. The biological treatment utilizes these microorganisms (fungi, bacteria, and actinomycetes) or biobased products (enzymes) to selectively resolve lignin and hemicellulose, which facilitates the process of enzyme digestion (Moodley et al., 2020). In addition, part of hemicellulose and cellulose would be consumed by microorganisms in the process of pretreatment. Furthermore, an efficient biological bacterium agent is necessary and a large sterile area should be retained during the whole biological pretreatment process (Canilha et al., 2012; Binod et al., 2016; Yu et al., 2019).

Fungi involve white-rot fungi, brown-rot fungi, and soft-rot fungi, and white-rot fungi are a physiologically different group of saprophytic fungi such as basidiomycetes and further cause white rot in wood (Akhtar et al., 2016). White-rot fungi pretreatment of lignocellulosic biomass is the best method of fungi treatment, which could remarkably degrade lignin under the function of the enzymes. Hence, white-rot fungi pretreatment plays an important role in fungal treatment. Lignin is entirely degraded into CO_2_ by white-rot fungi, revealing good performance in lignin removal. Moreover, diverse genera, species, and strains of white-rot fungi revealed more lignin removal. In general, lignin was attacked by white- and soft-rot fungi, while cellulose can be attacked by white-, brown-, and soft-rot fungi (Sun et al., 2011; Canilha et al., 2012).

Currently, numerous white-rot fungi have been studied on various straw biomass and revealed excellent delignification rates. Wheat straw was pretreated with 19 white-rot fungi, giving 35% reducing sugars under five-week pretreatment with *Pleurotus ostreatus*, while only 12% yield of reducing sugars was achieved from the untreated wheat straw. In addition, the application of five different fungi in wheat straw treatment was researched. The best production of overall sugars was with the treatment of *Aspergillus niger* and *A. awamori* (Talebnia et al., 2010). At present, numerous microorganisms should be investigated and improved in their capacity to undergo delignification through genetic engineering technology. For instance, *Saccharomyces cerevisiae* and *Escherichia coli* have been applied in the pretreatment of lignocellulosic biomass (Jönsson and Martín, 2016). Akyol et al. (2019) applied *Trametes versicolor* pretreatment on wheat, rye, and barely, achieving 80% cellulose degradation. Besides, wheat straw was pretreated with the *Ceriporiopsis subvermispora* strain for 10 weeks, and the digestibility and fermentable sugar were enhanced by 60 and 44% in comparison with the untreated one (Cianchetta et al., 2014).

Although biological treatment of lignocellulosic biomass could improve the saccharification efficiency, it requires a long reaction duration. For example, corn stover was pretreated by the white-rot fungus *Irpex lacteus* treatment for 42 days, obtaining 43.8% lignin removal, and the saccharification efficiency was sevenfold higher than the untreated one (Song et al., 2013). In addition, wheat straw was treated with *Ceriporiopsis subvermispora* pretreatment for 70 days, and the highest sugar yield was up to 44% (Cianchetta et al., 2014). However, combined with chemical or physical treatment, it could reduce the reaction time. The coupled pretreatment applying *P. ostreatus* for 18 days and 2% hydrogen peroxide for 48 h was more effective than a single treatment of rice hulls employing *P. ostreatus* for 60 days (Sindhu et al., 2016). An innovative strategy for the enhancement of treatment effectively applying a xylan-degrading microorganism could replace the conventional pretreatment of removing lignin with fungal. For instance, rice straw was pretreated with the cellulase-free enzyme–producing *Bacillus firmus* K-1 and its enzymes, and the results showed that 21% lignin was removed, 74% glucan was converted, and the cellulose crystalline and porosity were dramatically enhanced in comparison with those of the untreated rice straw (Baramee et al., 2020).

Fungi treatment needs to have long reaction duration (several weeks or months), while bacterial pretreatment requires less duration (a few hours) to be finished (Fang et al., 2018). At present, various bacteria (*Clostridium* sp., *Bacillus* sp., *Streptomyces* sp., *Thermomonospora* sp., *Cellulomonas* sp.,) have been broadly utilized in lignocellulosic biomass pretreatment (Sharma et al., 2019; Rezania et al., 2020).

Although biological treatment exhibits high saccharification efficiency and eco-friendliness, some shortcomings particularly the long pretreatment cycle are present. The treatment duration can be shortened by a combination of chemical and physical treatment techniques. Furthermore, the mechanisms of biological pretreatment are unknown attributes to the complex microorganism structure. Therefore, it is necessary to continue exploring the mechanisms of microorganism pretreatment.

## Combined Pretreatment

A single pretreatment faces many challenges, such as technical issues, the presence of pollution, higher energy inputs, longer reaction duration, anti-corrosion equipment, and difficulty to realize industrialization. Numerous investigations reported that a combination of physical, chemical, and biological pretreatments could appear more efficient than the single treatment method because the combined pretreatment revealed synergistic functions on the conversion of straw biomass and enzymatic hydrolysis (Sindhu et al., 2016). For instance, CO_2_ pretreatment is coupled with other methods such as ultrasound, alkaline hydrogen peroxide, ammonia explosion, steam explosion, and enzymatic hydrolysis, which can facilitate high hydrolysis efficiency and fermentable sugar yields (Table 4). Besides, CO_2_ pretreatment combined with enzymatic conversions becomes increasingly popular because of low temperature and safe solvents (Morais et al., 2015).

**TABLE 4:** Sc-CO_2_ combined with conventional treatment methods for biomass pretreatment.

Methods	Feedstocks	Pretreatment conditions	Conditions of conventional method	Glucose yield/reducing sugar yield (%)					Ref(s)
				Water content (%)	CO_2_ method	Conventional method	Both methods	Unpretreated biomass	
Steam^a^	Wheat straw	190°C, 120 bar, 60 min	200°C, 15 min	23	—	—	−/60.1	—	Yin et al. (2014)
Acetic acid/steam	Wheat straw	180°C, 180 bar, 45 min	180°C, 10 min (steam)	50	—	—	175^e^/-	—	Zabihi et al. (2021)
		180°C, 180 bar, 45 min	180°C, 45 min, 2 bar	50	—	—	275^e^/-	—	Zabihi et al. (2021)
Autohydrolysis	Wheat straw	210°C, 60 bar	—	—	2.62 CO_2_ mol-1	—	92	—	Pasquini et al. (2005)
AFEX	Rice straw	175°C, 7.5 bar, 30 min	15% NH_4_	—	46.75^b^/-	96.00^c^/-	99.04/-	—	Yin et al. (2014)
		165°C, 20 bar, 70 min	14.3% NH_4_	—	—	—	93.6	—	Cha et al. (2014)
Lime	Rice straw	pH 6	CaCO_3_	10	—	—	74	—	Silveira et al. (2015)
Ultrasound	Corn cob	170°C, 200 bar, 30 min	20 kHz, 600 W, 80°C, 6 h	50	31.0/62.0	—	42.0/87.0	10.0/12.5	Benazzi et al. (2013)
	Corn stalk	170°C, 200 bar, 30 min	20 kHz, 600 W, 80°C, 8 h	50	14.0^d^/25.5	—	16.0/30.0	13.5/16.6	Phan and Tan (2014)
Ultrasound	Sugarcane bagasse	80°C, 65 bar, 120 min	40 kHz, 154 W, 30°C, 8 h	65	-/380 ± 9^e^	-/350^e^	-/300^e^	-/127 ± 16^e^	Alinia et al. (2010)
		180°C, 206 bar, 60 min	35°C, 4 h	80	61.3/-	20.2/-	97.8/-	13.4/-	Phan and Tan (2014)
Alkaline/(H_2_O_2_/NaOH)	Sugarcane bagasse	180°C, 206 bar, 60 min	0.6% H_2_O_2_, 60°C, 9 h, CO_2_-assisted conventional methods	80	61.3/-	22.9/-	65.8/-	13.4/-	Cha et al. (2014)
Co-solvent (1-butanol/H_2_O)	Sugarcane bagasse	190°C, 70 bar, 105 min	60% 1-butanol	40	—	—	94.5^f^	—	Silva et al. (2013)

a CO_2_ explosion was performed after steam explosion.

b Conditions: 160°C, 50 min, 15 bar.

c Conditions: 160°C, 50 min, 10% ammonia concentration.

d CO_2_ treatment at 170°C and 200 bar of CO_2_ pressure for 1 h.

e Units of g·kg^−1^ of dry biomass.

f Units of delignification.

Single SE treatment could only degrade a major part of hemicellulose but not dramatically enhance the efficiency of lignin fractionation (Chen and Liu, 2015; Jönsson and Martín, 2016). Hence, it required assisting with other treatment technologies (LHW, Sc-CO_2_, ChCl) to obtain high-efficiency lignocellulose conversion. For example, SE pretreatment combined with LHW, IL, Sc-CO_2_, SO_2_-impregnated, wet oxidation, alkaline peroxide, and superfine grinding treatment could not only enhance the cellulose digestibility and hemicellulose degradation but also improve the fermentable sugar yields (Akhtar et al., 2016). For example, using wet explosion treatment of wheat straw achieved 70% cellulose and 68% hemicellulose, while 92–99% of lignin was extracted in combination with SE treatment (200–220°C, 15–22 bar, respectively) and alkaline peroxide treatment (2% H_2_O_2_, 50°C, 5 h, pH 11.5), as reported by Akhtar et al. (2016). Besides, using SO_2_-impregnated steam-exploded pretreatment of corn stover could obtain 89% glucose and 78% xylose (Akhtar et al., 2016). SE combined with choline chloride pretreatment on corn stover at 184°C, 1.0 MPa in 1:1.2 ratio for 15 min achieved 78.9% xylan, 74.6% glucan, and 84.7% lignin, respectively (Nasir et al., 2020). Overall, SE is an excellent treatment technology attributed to its numerous advantages such as high total solids, high recovery efficiency, high cellulose fractionation, low environmental pollution, low cost, and feasibility of industrialization. Therefore, it is a most promising technology combined with other approaches.

Biological treatment in combination with LHW, moderate physical, or chemical treatment is also reported (Sindhu et al., 2016). The major advantage is that fungal treatment combined with other technologies could decrease the operation time and increase the enzymatic hydrolysis yield, in comparison with the sole treatment (Rezania et al., 2020). For example, a combination of *Populus tomentosa* with LHW could obtain 92.33% hemicellulose removal and the highest glucose yield. In addition, the combined treatment using white-rot fungus *P. ostreatus* followed by AFEX treatment obtained higher fermentable sugars than the treatment of rice straw with the sole treatment of AFEX (Sindhu et al., 2016). Then, bacteria exhibited high efficiency for degrading lignin and increasing the enzyme digestion, which is an excellent selection for combining with other approaches. For example, bacteria (*Cupriavidus basilensis* B-8) treatment was combined with dilute acid pretreatment (H_2_SO_4_) of rice straw, and the enzymatic digestibility was increased by 70% compared to the sole dilute acid treatment (Yan et al., 2017). Furthermore, a combination of LHW treatment and disk milling treatment of sugarcane bagasse showed higher efficiency than single pretreatment of sugarcane bagasse using LHW. The combined treatment significantly enhanced glucose release by 41–177% under LHW at 140 –180°C for 10 min and then disk milled (Wang et al., 2018).

Alkali combined with microwaves could dramatically remove the lignin from the liquid biomass, while cellulose and hemicellulose were retained in the solid phase for further enzymatic digestion (Raman and Gnansounou, 2018). For instance, the microwave coupled with the alkaline treatment of wheat straw can enhance the treatment of efficiency, reduce the reaction time, and improve the delignification (Yu et al., 2010). Rice straw treated with 1% NaOH combined with acidified water wash at 121°C, 0.1 MPa for 30 min could obtain 80% cellulose and 65% lignin (Samar et al., 2020).

In addition, sugarcane straw was treated with dilute sulfuric acid (0.6% H_2_SO_4_) assisted by microwaves to enhance the yield of fermentable sugars and minimize the concentration of inhibitors as well as reducing the time consumption (Fonseca et al., 2021). As a novel approach for pretreating straw biomass, 85% H_3_PO_4_ plus 30% H_2_O_2_ was applied as a treatment solvent to fractionate wheat straw. The accessibility and enzymatic hydrolysis of cellulose were highly increased (Wan et al., 2018). Corn stover was pretreated with two-stage dilute hydrochloric acid (DA)/aqueous ammonia wet oxidation (AWO), obtaining 82.8% xylan in the first stage at 120°C for 40 min with 1 wt% HCl, achieving 86.1% lignin removal in the second stage at 130°C for 40 min with 12.6% ammonium hydroxide and 3.0 MPa O_2_, and achieving 71.5% glucan with a low enzyme dose (An et al., 2019).

Ultrasound-assisted DES treatment could be an effective treatment approach for straw biomass. For example, 36.7% reducing sugar was obtained from oil palm empty fruit bunch (OPEFB) under ChCl:LA coupled with 60% sonication power (210 W) at 50°C for 30 min (Lee, et al., 2021). Furthermore, corn straw was pretreated in combination with ultrasonic [20 k (60 W) and 40 k (60 W) for 30 min], microwave (120°C, 1 min), and DES (ChCl:OA:Gly) treatment, achieving 61.5% lignin, 90.3% hemicellulose, and 76.1% cellulose (Yan et al., 2021). Microwaves could maximize the ionic character of DESs and improve their molecular polarity as well as maintaining lower treatment time and temperature. For example, wheat straw was pretreated combining the microwave treatment (360 W, 8 min) with DESs (ChCl:FA = 1:3), obtaining maximum total sugars (619 mg/g) being twice in comparison with those in the single DES treatment (Isci et al., 2020). At present, few investigations had reported on the effect of joint treatment to assist NADESs; therefore, coupling other technologies with NADES treatment is a promising strategy for pretreatment of straw biomass.

There are some disadvantages of IL treatment such as redeposition of lignin onto the surface, which could obstruct the accessibility of enzymes and reduce the effective hydrolysis. Technologies like combinatorial utilization of ILs with other treatment surfactants could elevate the treatment efficacy and decrease the whole process cost, owing to that the surfactants could prevent the lignin redeposition on the surface of polysaccharides. For example, in the combination treatment of *Saccharum spontaneum* biomass (SSB) with tris(2-hydroxyethyl)methylammonium methylsulfate ([TMA][MeSO_4_]) and sodium dodecylsulfate (SDS), following its enzymatic hydrolysis under consolidated bioprocess (CBP), the sugar yield was increased by 2.35-fold in comparison with the untreated one (Vaid et al., 2021). Moreover, *Miscanthus* hybrid (*Mx*27999) was pretreated by SE coupled with ILs and single treatments for the generation of oligosaccharides, respectively (Bhatia et al., 2021).

Diverse treatment technologies are applied to disrupt the strong natural recalcitrance of straw biomass by identifying the limiting factors on enzymatic digestion. There are various criteria for efficient methods that are as follows: 1) reducing the degradation of hemicellulose and keeping a high sugar content, 2) minimizing the energy consumption, 3) decreasing the harmful side products, and 4) an eco-friendly, cost-effective, and mild reaction process (Nasir et al., 2020). According to investigations, utilizing microwaves and ultrasound for the efficiency of the treatment process under mild conditions exhibited a decrease in delignification. Hence, balancing the harshness of treatment conditions and the effectiveness of hemicellulose, cellulose, and lignin separation is still a big challenge.

The major obstacle of straw biomass application is the complex structure and heterogeneity of lignocellulose. Although various approaches (chemical, physical, biological, and combined treatments) have been employed for lignocellulose pretreatment, the lignin fractionation efficiency is the main challenge. Achieving the high-efficiency conversion of biomass depends on the effectiveness of lignin fractionation and modification. For improving selective delignification, various technologies have been reported. For instance, LHW combined with several metal oxides (MgO, ZnO, CuO), or diluted peracetic acid could enhance the efficiency of lignin removal, with less fermentable sugar loss (Li et al., 2018; Choi et al., 2019). In addition, to achieve high-purity lignin, organosolv pretreatment could be coupled with SE treatment (Matsakas et al., 2019). Then, applying a biphasic system consisting of H_2_O and oxalic acid could obtain high lignin removal attributed to decrease the lignin condensation and repolymerization reactions (Li et al., 2017). Therefore, only clearly understanding the lignin properties (content, condensation degree, molecular weights, S/G/H ratio, the number of phenolic hydroxyl groups) can explore the optimum pretreatment strategy for overcoming the lignin recalcitrance and achieving the ideal conversion results.

In a brief summary, the combined pretreatment methods are more effective than chemical or biological technology alone. However, the major components (hemicellulose, cellulose, and lignin) of diverse straw reveal a distinct difference. Therefore, the efficiency of conversion and saccharification of straw biomass is dependent on the raw materials. To achieve the best results of straw pretreatment, an appropriate technology should be chosen according to various straw biomass resources. At present, no single pretreatment technology can fully realize the economic, environmental-friendly, and efficient treatment of biomass. Although the combined pretreatment has achieved some satisfactory results, it is still necessary to further develop the combined treatment technology to explore its full potential and realize the efficient biomass pretreatment (Kumar and Sharma, 2017).

## Pretreatment Methods for Converting Straw Into Fermentable Sugar

Using straw biomass to generate valuable chemicals and biofuels involves some key processes including saccharification and fermentation and further conversion, while the lignin is separated from the solid residue and the final products are purified. Only solving the recalcitrant problem of polysaccharides in biomass can make full utilization of polysaccharides in the straw biomass (Qi et al., 2018). There are plenty of factors influencing pretreatment efficiencies, such as the type of biomass, various reactors, and diverse reaction conditions (Zhang et al., 2015; Carvalho et al., 2016). The transformation of straw to fermentable sugars is a very complex process (Singh et al., 2014; Qi et al., 2018; Raman and Gnansounou, 2018; Yuan et al., 2018). The fermentation and saccharification of lignocellulosic materials with pretreatments approaches could obtain 90% aggregate sugars. In sharp contrast, without any pretreatment technique, only lower than 20% sugars are achieved (Valaskova et al., 2007; Ponnusamy et al., 2019). Hence, choosing an appropriate pretreatment technology would accord to the unique feature of feedstock (Raman and Gnansounou, 2018; Yuan et al., 2018). The advantages and disadvantages of diverse treatment approaches are summarized in Table 5.

**TABLE 5:** Advantages and disadvantages of diverse treatment approaches.

Treatment approaches		Advantages	Disadvantages
Chemical pretreatment	CO_2_ explosion pretreatment	Low cost, low temperature, high solid loading, enhances the accessible surface area, and does not form toxic compounds	High pressure, high requirement of equipment
	Oxidative pretreatment	Removes lignin effectively, environmental-friendly, less side products	High cost, difficult to separate the solvents
	Steam explosion pretreatment	Applies no chemicals and less H_2_O, low cost, and low environmental pollution	High pressure and temperature
	Supercritical fluid pretreatment	Uses green solvents, does not degrade sugars, and suitable for mobile biomass processor	High cost
	SO_2_ explosion pretreatment	The solubilization of hemicellulose through adding the external acid provides partial cellulose hydrolysis and requires low temperature	Stringent equipment and inhibitory compounds when using acids
	Ammonia fiber explosion	Removes lignin efficiently, enhances enzyme accessibility, reduces the formation of inhibitors, and needs fewer enzymes	Expensive separation and recycle, not efficient for biomass with high lignin content
	Liquid hot water	Obtains pure hemicellulose, does not add chemicals or catalyst, hydrolyzes hemicellulose, achieves a high yield of sugars, and does not require washing, recovery, and detoxifying	Requires high energy
	Alkali pretreatment	Low temperature and pressure, low carbohydrate degradation, low corrosion, lignin removal, low cost	Longer residence times, generation of salt needs to neutralize and recycle, high consumption energy
	Acid pretreatment	Concentrated acid: high hemicellulose solubility, positive effect on cellulose enzyme digestion, and high yields of glucose. Dilute acid: low cost, effective, does not require recycling acid, and high enzymatic digestibility	Highly toxic, corrosive, high temperature and pressure, produces inhibitors, requires expensive materials, catalyst recovery problem, environmental problem, needs neutralization and detoxification
	Ionic liquid pretreatment	Less energy, easy to operate, conducted in pilot scale	Expensive, high viscosity, high cost of recovery and recycling
	Deep eutectic solvents	Green solvents, biodegradable and biocompatible, highly tunable, convenient synthesis	Hygroscopicity, instability, and high viscosity
	Natural deep eutectic solvents	Green solvents, consist of certain natural compounds, environment friendly	High viscosity
	Organosolv pretreatment	Hydrolyzes hemicellulose and lignin and achieves high-purity lignin	High cost of recovery and reuse, high inhibitors, environmentally unfriendly, low biomass recovery rate
	Sulfite pretreatment	Removes lignin, energy-efficient	Reduces biomass size
Physical pretreatment	Microwave pretreatment	Short time, energy-efficient, simple operation, non-polluting, selective degradation of hemicellulose and lignin	High cost and long reaction time leading to slow production
	Electron beam irradiation	Mainly effective on depolymerizing cellulose, improves the surface area, does not form inhibitors, cost-effective	Does not affect hemicellulose and lignin, high pressure, less efficient
	Ultrasonic pretreatment	Enhances reactivity and accessibility of cellulose	Having a negative impact on enzymatic hydrolysis
	Pyrolysis pretreatment	Degrades cellulose quickly	High cost, low yield
	High-energy electron radiation pretreatment	Decreases cellulose polymerization degree	High cost
	Mechanical comminution	Decreases cellulose crystallinity and particle size and does not form inhibitors	Cannot remove hemicellulose and lignin, high energy, and low conversion efficiency
Biological pretreatment		Degrades hemicellulose and lignin selectively, low-energy input, does not add catalyst or chemicals, does not form toxic compounds, cost-effective, environment friendly	Low enzyme digestion, long incubation time, slow reaction process, low downstream yields, high sensitivity to inhibition
Combined pretreatment		Reveals combined actions on saccharification and fermentation processes	Need to overcome the drawbacks of every single treatment

Currently, diverse pretreatment methods such as biological, physical, and chemical approaches have significantly improved the yields of fermentable sugars from the conversion of polysaccharides (Zhang et al., 2015; Agudelo et al., 2016; Qu. et al., 2016; Carvalho et al., 2016; Qi et al., 2018; Raman and Gnansounou, 2018; Satari et al., 2019). The abovementioned technologies have some connections and distinctions. Firstly, all these techniques have positive effects on lignocellulose depolymerization by altering the particle size, biomass recalcitrance, structure, and chemical components of straw biomass. For example, biological treatment has its characteristics including cost-effectiveness, eco-friendliness, and high degradation efficiency of straw materials (Valaskova et al., 2007). Physical pretreatment methods have positive effect s on the efficiency of fermentation, saccharification, and hydrolysis and the formation of valuable organic chemicals (Kucharska et al., 2018). Besides, employing physical pretreatment of straw biomass could decrease the amounts of subproducts produced during fermentation and hydrolysis (Abbasi and Abbasi, 2010). Chemical pretreatment applying many chemicals such as acids, alkalis, organic solvents, and ionic liquids could bring an obvious positive influence on the native structure of straw materials (Ponnusamy et al., 2019). Furthermore, the chemical pretreatment of straw biomass exhibits excellent conversion efficiency and requires a short duration (Singh et al., 2014).

Secondly, these pretreatment methods have unique characteristics. For instance, biological treatment usually employing microorganisms to decompose lignin via enzymes or chemical approaches make the lignocellulosic materials easier to undergo saccharification through the polysaccharide sugar units (Valaskova et al., 2007). The most common microorganisms are white fungi. Now, almost 51 white-rot basidiomycetes of *Punctularia* sp. are available (Ponnusamy et al., 2019). Besides, the efficiency of physical treatment is mainly improved by reducing the particles, the size of feedstocks, the degree of polymerization, and the crystallization of the straw biomass (Sun and Cheng, 2002). Physical treatment involves extrusion, milling, and sonication methods. Extrusion is a common approach, which is combined with other technologies to enhance treatment efficiency. For instance, the extrusion treatment coupled with steam explosion pretreatment of barley straw gives 84% glucans, 91% hemicellulose, and 87% lignin, respectively (Oliva et al., 2017).

Among the chemical pretreatment approaches, dilute acid and alkaline pretreatments are favored. In addition, the dilute pretreatment technique is an excellent method with low-lignin content lignocellulose (Ponnusamy et al., 2019). For instance, applying 2% H_2_SO_4_ at 120°C for 43 min of corn straw obtained 77% xylose (Liu et al., 2003). Approximately 50% lignin and nearly 100% hemicellulose were hydrolyzed by 2% H_2_O_2_ at 30°C for 8 h, achieving 95% glucose (Ponnusamy et al., 2019). In addition, organosolv treatment could completely solubilize hemicellulose and extract lignin. The method is indeed effective and, nevertheless, mainly employs the chemicals that need an extra step for solvent recycling (Jędrzejczyk et al., 2019). Oxidative pretreatment is another type of chemical treatment, which is not common because of high cost and energy consumption (Cheah et al., 2020).

For chemical pretreatment, the steam explosion treatment utilizes 70% less energy in comparison with physical treatments. Furthermore, hemicellulose is hydrolyzed and acids are generated *in situ*, further decomposing the hemicellulose. Nevertheless, the design of reactor equipment needs to be optimized (Singh et al., 2014). Ammonia fiber explosion is nearly similar to steam explosion. However, the major obstacle is the cost of ammonia and its recovery (Cheah et al., 2020). The hot water treatment approach does not utilize any chemicals and has no requirement of applying the anti-corrosion reactor but forms few toxic components (Jiang et al., 2015).

In general, biological pretreatment technology is more sustainable and environmentally friendly in terms of producing fermentable sugars, but it requires a much longer time to treat straw biomass. In sharp contrast, chemical treatment techniques, such as inorganic acids (sulfuric acid), are economically viable while causing environmental pollution. Although chemical pretreatment exhibits high efficiency, it requires rigorous reactors, high cost, and high-energy inputs. The single physical treatment is difficult to commercialize because of high cost and high-energy inputs. Consequently, combined pretreatment technologies have better efficiency and are more sustainable than any other single method, while plenty of studies are still required to make full use of the advantages and potential of combination treatment approaches.

## Conclusion and Future Outlook

The main bottleneck in pretreatment technologies for straw biomass is the presence of lignin in the feedstocks that could drastically affect the enzymatic digestion of hemicellulose and cellulose. Therefore, the delignification of straw biomass is the crucial step in the extensive studies in the development of diverse treatment processes. To date, a single treatment technique has not been realized for delignification without sugar degradation. Although combined treatment approaches have obtained some satisfying results to an extent, still numerous extensive studies have to be further researched and investigated for improving lignin removal and fermentable sugar yield in an economic and green manner. Hereby, some investigation prospects are proposed as follows.

Fundamental researches on a structural and molecular level of lignocellulosic biomass should be reinforced. Hence, it requires combining with the different research fields to change the fundamental structure of biomass and further improve the pretreatment efficiency. Therefore, pretreatment technologies should not only focus on cellulose enzyme digestion, sugar yields, and the removal rate of lignin apparent indexes but also aim to research the mechanism theoretically involved—physical, chemical, or biological—in the transfer and reaction processes. It is indispensable to investigate the components and structure of various straw biomass and further study the influence of lignocellulosic structure on the ratio of conversion and enzymatic hydrolysis during processing.

Furthermore, the unknown inhibitory components in pretreated feedstocks should be identified and characterized in the whole pretreatment process, to decrease the expenses of treatment, fermentation systems, and reactor configuration. Optimizing the pretreatment approaches can promote the efficiency of combining with saccharification and fermentation, with close attention on the efficient feasibility for the commercial-scale biorefinery. Most importantly, the pretreatment approach should be optimized in terms of energy input and eco-friendly process.

Computational tools have become more and more promising and attractive to analyze the chemical processes automatically and identify the optimum experiment methodologies quickly. Hence, it is very important to apply computational tools to construct the process modeling and simulation for optimizing the economic efficiency of the biomass pretreatment process, for instance, pyrolysis process kinetic models, xylan degradation kinetics, enzymatic saccharification optimization, modeling mass flow and reaction temperature, and time of the treatment process (Seidl and Goulart, 2020).

Novel treatment approaches are required to be explored to promote the efficiency of lignocellulosic biomass conversion, saccharification, and fermentation. One optimum pretreatment approach is not possible for every type of lignocellulosic biomass attributed to the different content of hemicellulose, cellulose, and lignin for various biomass because the component could vary with a different plant or species or within species depending on their environment and source. Furthermore, every treatment approach has its unique properties and is utilized to a certain kind of biomass. Therefore, the reported pretreatment technology only demonstrated that it was an appropriate method for the special biomass, not other types of biomass. It is necessary to further investigate the optimum treatment methods for the different biomass according to its type, source, structure, and composition. Besides, computational technologies are required to be employed to optimize the lignocellulosic biomass pretreatment.

Existing open literature refers to the non-conventional or emerging methods like non-ionizing and ionizing radiation, high pressure, and pulsed-electric field. Yet detailed research is needed for further studying the reaction mechanism of diverse straw biomass treatments by employing non-conventional energies. Sustainable, less energy-consumed, capital cost–minimized, environmentally benign, economic, and efficient pretreatment technologies are still challenges of industrial scale-up pretreatment of straw biomass.
